# Characterization of the Active Microbiotas Associated with Honey Bees Reveals Healthier and Broader Communities when Colonies are Genetically Diverse

**DOI:** 10.1371/journal.pone.0032962

**Published:** 2012-03-12

**Authors:** Heather R. Mattila, Daniela Rios, Victoria E. Walker-Sperling, Guus Roeselers, Irene L. G. Newton

**Affiliations:** 1 Department of Biological Sciences, Wellesley College, Wellesley, Massachussetts, United States of America; 2 Microbiology & Systems Biology group, TNO, Utrechtseweg, Zeist, The Netherlands; 3 Department of Biology, Indiana University, Bloomington, Indiana, United States of America; Arizona State University, United States of America

## Abstract

Recent losses of honey bee colonies have led to increased interest in the microbial communities that are associated with these important pollinators. A critical function that bacteria perform for their honey bee hosts, but one that is poorly understood, is the transformation of worker-collected pollen into bee bread, a nutritious food product that can be stored for long periods in colonies. We used 16S rRNA pyrosequencing to comprehensively characterize in genetically diverse and genetically uniform colonies the active bacterial communities that are found on honey bees, in their digestive tracts, and in bee bread. This method provided insights that have not been revealed by past studies into the content and benefits of honey bee-associated microbial communities. Colony microbiotas differed substantially between sampling environments and were dominated by several anaerobic bacterial genera never before associated with honey bees, but renowned for their use by humans to ferment food. Colonies with genetically diverse populations of workers, a result of the highly promiscuous mating behavior of queens, benefited from greater microbial diversity, reduced pathogen loads, and increased abundance of putatively helpful bacteria, particularly species from the potentially probiotic genus *Bifidobacterium*. Across all colonies, *Bifidobacterium* activity was negatively correlated with the activity of genera that include pathogenic microbes; this relationship suggests a possible target for understanding whether microbes provide protective benefits to honey bees. Within-colony diversity shapes microbiotas associated with honey bees in ways that may have important repercussions for colony function and health. Our findings illuminate the importance of honey bee-bacteria symbioses and examine their intersection with nutrition, pathogen load, and genetic diversity, factors that are considered key to understanding honey bee decline.

## Introduction

Recent challenges to honey bee health, including dramatic colony losses attributable to Colony Collapse Disorder (CCD) and the introduction of pests and pathogens into managed colonies, have devastated honey bee stocks worldwide [1], [2]. However, a causative agent has yet to be identified [3]–[5] and new ideas about factors that might explain a decline in the health of honey bee colonies are still emerging [6]. At present, honey bee researchers view these alarming losses as a likely product of multiple honey bee pathogens overlapped with chronic stressors, including poor nutrition, increased pathogen loads, and a lack of genetic diversity among colonies' work forces [2], [7]–[15]. One factor that is likely shaped by colony genotype and is critical for easing nutritional stress—but has not yet been fully characterized—is the composition and function of honey bee microbiotas. The breadth of bacterial flora (and other microbes) that are found in honey bee colonies may play a role in the health and vitality of these organisms, much as they do in our own bodies [16]. Host-associated microorganisms contribute enormously to the development of their host's immune system, digestion, and general well being [17]–[19].

Many animals coexist with bacterial symbionts that make available to their hosts nutrients that are either absent from their host's diet or otherwise unavailable to them in the foods that they consume [20], [21]. Symbioses of this nature are especially critical to animals with plant-based diets because most of them do not produce enzymes that digest plant cellular material (including lignin and complex polysaccharides), whereas many bacterial species do [22]–[25]. Honey bees (*Apis mellifera*) have a diet that consists entirely of foods that are derived from plants: nectar and pollen. Bees process nectar into honey, which provides the colony with their primary source of carbohydrates and holds only trace amounts of amino acids and vitamins [26]–[29]. Pollen provides honey bees with virtually all of their remaining nutrients, including amino acids, lipids, vitamins and minerals [30], [31]. However, the cytoplasmic nutrients in pollen are not readily available to bees because each pollen grain has a cell wall that is chemically difficult to degrade (e.g., an extremely resistant sporopollenin outer layer underlain by a layer of cellulose). Honey bees are one of the few insects known to have genes that encode cellulases [32], but their persistent difficulty with pollen digestion is evidenced by the substantial proportion of pollen grains that are not fully broken down in the guts of workers [33]. Furthermore, most pollen sources do not provide a complete complement of the nutrients that honey bees require or may contain only trace amounts of some essential amino acids [34]–[36], which means that bees must collect a mix of pollen types when they can.

To alleviate some of these nutritional challenges, honey bees typically do not consume raw pollen. Instead, workers process pollen that they collect by packing it into honeycomb, adding glandular secretions to it, and sealing it with a drop of honey [37]. Pollen processed in this way is matured into bee bread after several weeks, presumably due to the activity of microorganisms that are found in bee bread, but are absent in unprocessed pollen [38]. Bee bread is chemically different from pollen: it has a higher vitamin content [39], lower amounts of complex polysaccharides, a shift in amino acid profile [40], and lower pH [41], [42]. It is routinely suggested that these changes in nutritional composition are a result of the metabolic activity of the microflora that is present in stored pollen [37], [38], [41], although the organisms that are actively involved in this metabolic transformation have never been definitively identified. Previous studies have characterized bacteria that are associated with bees using culturing techniques alone [40], [43]–[45] or culture independent approaches such as 16S rRNA gene cloning and sequencing [4], [46]–[48]. Culture-based studies provide an important perspective on the microbiotic world of honey bee colonies, but they necessarily preclude the vast majority of bacteria, which are unculturable. Culture-independent studies have added to this perspective, but they have been relatively small in scope (both in terms of bee sampling and 16S rRNA gene sequencing) and have not differentiated between bacteria that *actively* transform pollen to bee bread versus those that are merely present in it. Although the organisms that are responsible for this conversion have remained largely a mystery, it is clear that bee bread is more nutritious to workers than unprocessed pollen. Honey bees fed the former food live longer than those that are fed the latter [49] and are better able to offset physiological damage from pests when bee bread is abundantly available [50]. Because of the way that bee bread is inoculated, matured, and distributed, its microbial community acts as an extended gut for the colony, and the benefits of its activity are shared amongst all colony members.

One way that the breadth and activity of a colony's microbiota may be enhanced is through an increase in the genetic diversity of its worker population. Unlike queens of most social hymenopteran species (bees, ants, and wasps), a honey bee queen mates with a large number of males and therefore introduces into her colony genetically diverse families of workers from many different fathers. In *A. mellifera*, each queen mates with an average of 12 males [51], with a reported record of 44 mates [52]. Extreme polyandry on the part of queens is a highly derived trait, but one that is found universally in the honey bee genus *Apis* [53] and to a similar degree among a limited number of other social insect taxa, including army ants [54] and leaf-cutter ants [55]. Honey bees benefit from the high level of within-colony genetic diversity that extreme polyandry generates through an increased ability to mitigate symptoms of pathogen and parasite infection [56]–[58] and higher levels of colony stability [59] and productivity [59]–[65]. These studies suggest that there are plural reasons why extreme polyandry and the within-colony genetic diversity that it generates have been universally selected for in honey bees. Given the scientific consensus that the genetic background of an animal significantly impacts the composition of its microbiome [18], [66]–[70] it is possible that genetic diversity in a honey bee colony may also foster a more diverse bacterial flora, which may in turn confer either a protective or a nutritional advantage to all colony members.

With the link between colony health, productivity, and nutrition in mind, we aimed in this study to describe the *active* bacterial microbiotas that are associated with honey bees and their food products by making two central queries. Firstly, we characterized the composition of bee-associated bacterial communities to illuminate the role that active microbes play in maturing pollen into bee bread and the relevance of their activity to bee nutrition. Additionally, we investigated whether increased genetic diversity within a colony's worker population translated into changes in the diversity and composition of microbes that were associated with bees and their food. To answer these questions, we utilized barcoded amplicon pyrosequencing—a deep-sequencing, culture-independent approach to analyzing microbial diversity—to explore differences in bacterial communities in honey bee colonies that had either a high level of genetic diversity, characteristic of naturally occurring *Apis*, or a low level of within-colony diversity, like that produced by ancestrally monoandrous bee queens [71]. We chose to query the active microbial communities in honey bee colonies by beginning our analysis with total RNA, an approach that has never before been used to examine bees and their symbiotic microbes. Accordingly, our findings differ in substantial ways from previous reports about the microbes that are associated with honey bees because the largest fraction of active microbes reported herein had not been identified by other authors.

## Results

### Active microbiotas differ greatly across environments in honey bee colonies

**Figure 1:**
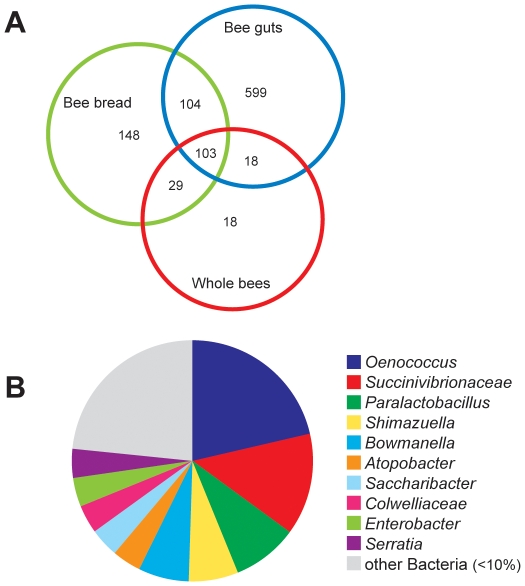
Diversity of species and genera found within different honey bee colony environments. (A) Venn diagram representation of species-level diversity (97% identity) of the active bacterial communities that were found within three bee-associated sampling environments (bee bread, bee guts, and whole bees), pooled across colony type. The total species richness in the dataset was 1,019 OTUs, with the most species-rich environment being bee guts (824 total species). (B) The core microbiota among all three environments included 103 species that spanned 26 genera, with *Oenococcus* and *Succinivibrionaceae* comprising the largest fractions.

**Figure 2:**
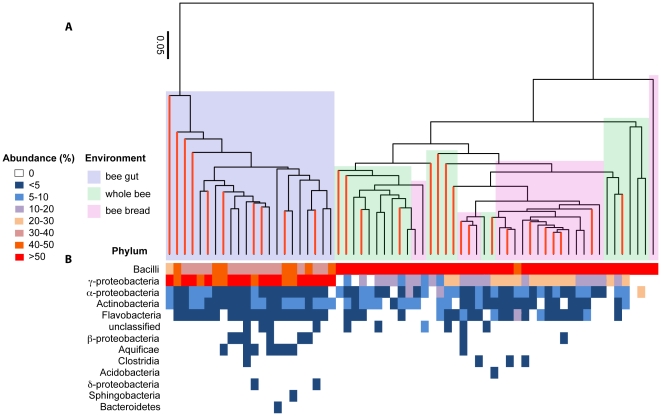
Honey bee colony samples cluster according to environment sampled. (A) Weighted, species-based (97% identity) Unifrac clustering of sampled environments in each study colony, with clades colored coded by environment. Additionally, branches representing the microbiota found in genetically uniform colonies are colored in red; black branches are genetically diverse colonies. (B) Each column below a Unifrac tree tip is the ranked abundance of bacterial classes found that sample, represented as a heat map; the most active classes were Bacilli and γ-proteobacteria. Bee-gut samples (in lavender) cluster to the exclusion of whole-bee (in green) and bee-bread samples (in pink), largely because of the presence of *Succinovibrionaceae*.

After all data were processed, we had a total of 70,562 high-quality, aligned pyrosequences that were subsequently classified to bacterial genera. The majority of these pyrosequences (56,556) were from bee guts, which allowed us the ability to detect the “rare biosphere” within this complex community. The balance of the pyrosequences was split between whole bees (4,471) and bee bread (9,535) and, although relatively smaller than the pool of sequences from bee guts, our dataset was large enough across all three sampling environments to permit comparison of the composition of their most active bacterial members. A total of 1,019 species belonging to five phyla were found across all bee-gut, bee-bread, and whole-bee samples, with the dominant phylum based on counts in all samples being Firmicutes (Table 1). Stored pollen is presumed to be inoculated with bee-associated microbes prior to maturation into bee bread [72] and our results suggest that both bee bread and bee guts contain 207 species mutually (Figures 1, 2). However, a substantial percentage of the species found in bee guts were not found in bee bread, and vice versa (75% and 46%, respectively; Figure 1). A “core” colony microbiome of 103 species was identified across sampling environments (Figure 1), which means that they shared only 10% of identified species in common. When samples were examined to determine how they grouped based on species diversity and abundance (defined by Unifrac clustering based on weighted species abundance), bee-gut samples clustered to the exclusion of whole-bee and bee-bread samples (Figure 2), which suggests that internal, active microbiomes (i.e., inside digestive tracts) were characterized by a different microbiota than environments that included external colony surfaces (i.e., bee bread and whole bees, which are less easily discriminated from one another). These results were found to be independent of library size; we obtained a similar outcome when all libraries were scaled to the same size (using the sub.sample function in Mothur) compared to use of entire libraries (Figures S2 and S3). For that reason, we discuss only the full dataset going forward. We identified a greater number of active bacterial species in bee guts than in whole bees, which at first glance is a counterintuitive finding (i.e., species detected in guts are expected to be found in whole-bee samples). However, this observation is likely a consequence of much deeper pyrosequencing of bee guts compared to whole-bee samples because rare species (i.e., singletons and doubletons) accounted for much of the extra species-level diversity that was found in the bee gut (see Figure S1). We assume that whole-bee samples are more complex (including many different niches and microenvironments) and the number of pyrosequences necessary to be able to sufficiently sample this environment to detect the “rare biosphere” was not achieved. However, the most active members in all three colony environments could be compared and were dominated by four phyla (Table 1; further characterized in the next section) whose bacteria are found in other animal guts: Proteobacteria, Firmicutes, Actinobacteria and Bacteroidetes [67].

**Table 1:** Sequence abundance of the 13 most active taxa from each colony environment that affiliated with distinct phylogenetic groups.

Genus/Family	Phylum	Bee guts	Bee bread	Whole bee
*Succinivibrionaceae*	Proteobacteria	38.8%(148)	0.16% (45)	0.04% (24)
*Bowmanella*	Proteobacteria	14.3% (149)	0.06% (45)	0.02% (24)
*Oenococcus*	Firmicutes	14.1% (101)	27.5% (56)	32.6% (24)
*Paralactobacillus*	Firmicutes	10.2% (84)	24.9% (51)	28.0% (20)
*Colwelliaceae* 1	Proteobacteria	6.4% (39)	0.02% (6)	0.00% (0)
*Bifidobacterium*	Firmicutes	4.7% (20)	0.72% (14)	2.0% (5)
*Shimazuella*	Firmicutes	3.2% (63)	11.4% (17)	14.1% (10)
*Enterobacter*	Proteobacteria	1.2% (11)	2.7% (4)	3.4% (5)
*Laribacter*	Proteobacteria	1.0% (20)	0.84% (8)	0.67% (3)
*Saccharibacter*	Proteobacteria	0.92% (21)	9.1% (8)	6.7% (9)
*Rummeliibacillus*	Firmicutes	0.52% (11)	2.1% (6)	2.0% (2)
*Atopobacter*	Firmicutes	0.28% (11)	1.76% (8)	2.4% (4)
*Escherichia/Shigella*	Proteobacteria	0.17% (6)	1.37% (2)	1.25% (2)
Others		4.2%	17.3%	6.8%

1 unclassified (could not classify beyond family for this group).

Percentage of total sequences (classified into genera) are given, as well as the number of species (based on 97% sequence identity) that was found within each genus in parentheses. See supplementary materials for a complete list of all bacterial sequences from the sampled environments from each study colony (Tables S2, S3).

### Genera that are most active in honey bee colonies are familiar from other anaerobic fermentation environments

Microbes associated with fermentation of human-produced foods and fermentation in other habitats comprised a substantial fraction of honey bee microbiotas, but had not been identified previously in colonies. *Succinivibrio* (associated with cow rumens), *Oenococcus* (important for wine fermentation), *Paralactobacillus* (important in food fermentation), and *Bifidobacterium* (associated with yogurt) were in the top-six most active genera found in bee-gut samples and accounted for more than 67% of the active bacterial community in that environment (Table 1). *Oenococcus* and *Paralactobacillus* were the most active microbes in bee bread and in whole bees, comprising 52% and 60% of bacteria represented in those communities, respectively (Table 1). Of the18 species in bee-bread samples that each made up at least 1% of the active bacterial community, 17 of these species were facultative or obligate anaerobes (Table S1). These species included many lactic acid bacteria (LABs; *Oenoccoccus*, *Paralactobacillus*, *Bifidobacterium*) as well as enterics (*Enterobacter*, *Escherichia/Shigella*, *Klebsiella*, and *Serratia*). The overwhelming activity of anaerobes associated with bee bread and bee guts suggests that their presence may be critical for converting pollen into a bee-bread food product that is suitable for long-term storage in colonies.

*Succinivibrionaceae* are γ-proteobacterial obligate anaerobes not previously known to associate with honey bees, but we found them to be extraordinarily active in bee guts, (although they were undetected in both bee-bread and whole-bee samples). Isolates from the genus *Succinivibrio* are known from cow rumens, where they play a role in the digestion of starches and the production of organic acids [73]. The active presence in bee guts of organisms from this genus largely caused this microenvironment to cluster to the exclusion of the other colony environments that were sampled (Figure 2). The importance of the *Succinivibrionaceae* for separating bee-gut microbiotas from microbiotas in other colony samples is supported by a significant and positive relationship between the relative activity of *Succinivibrionaceae* and the first component of a Principle Component Analysis (PCA) that determined whether bee-associated environments clustered based on the diversity and activity level of bacterial species that were present in them (Pearson correlation: r = −0.96, n = 64, p<0.0001). Organisms identified as *Succinivibrionaceae* in bee samples were 80–90% identical to known isolates *S. dextrinosolvens* and *S. amylolytica*. Interestingly, sequences from bee guts that were classified as *Succinivibrionaecae* were extraordinarily diverse and included 148 different species across all gut samples (Table 1).

*Oenococcus*, another genus not previously known to be associated with honey bee colonies, was the second most common genus in bee-gut samples and the most common one found in bee-bread and whole-bee samples (Table 1). *Oenococcus* also formed a large fraction (21%) of the core microbiota that was active in all sample types (Figure 1B), suggesting that it plays a significant role in the microbiome of honey bee colonies in general. *Oenococcus oeni*, the only characterized member of this genus, is a facultatively anaerobic lactic acid bacterium that is well known for its participation in malolactic fermentation of wine [74]. *Oenococcus* utilizes hexoses and pentoses as carbohydrate sources, including cellobiose, the disaccharide component of cellulose [74]. Species affiliating with *Oenococcus oeni* in our analysis ranged in their sequence identity relative to known isolates (77–80% nucleotide identity) and may represent a novel group of organisms within the *Oenococcus*, *Leuconostoc*, and *Weissella* clade.

**Figure 3:**
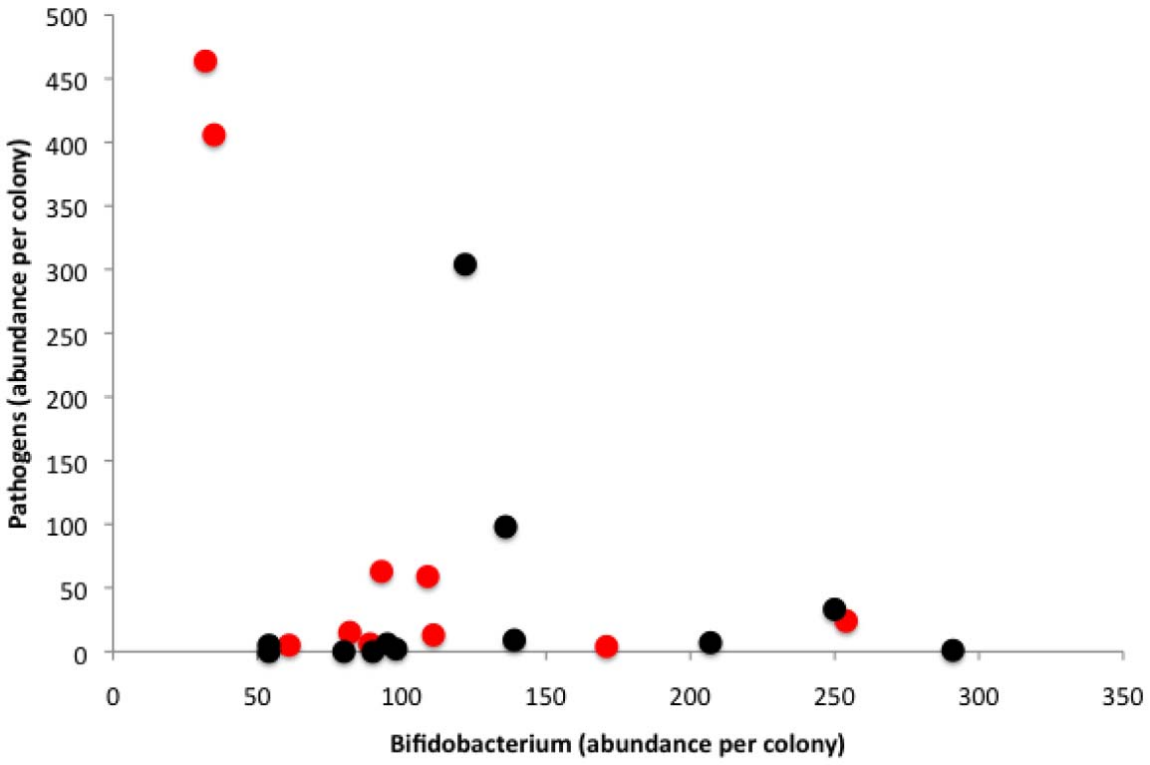
*Bifidobacterium* abundance inversely correlated with pathogen abundance. Abundance per colony of sequences of active *Bifidobacterium* (a known probiotic) was significantly and inversely correlated with the sequence abundance in the bee gut of species belonging to known pathogenic genera (Pearson correlation: r = −0.41, n = 22, p<0.05). Each data point represents a single study colony, with genetically uniform colonies (red) distinguished from genetically diverse ones (black).

*Bifidobacterium* and *Paraloctobacillus*, two potentially probiotic genera whose members are famous for their involvement in food fermentation [75], [76], were found to be highly active in every colony that was sampled (Table 1). *Paralactobacillus* was part of the core colony microbiota (Figure 1B) and comprised 10–28% of the total microbiota in each sample type (Table 1). *Bifidobacterium* was a smaller fraction of the active community across each colony environment and accounted for <1 to 4.7% of total sequences. Interestingly, the load of genera affiliated with plant and animal pathogens that were active in bee guts (Table 2) was negatively correlated with the activity in guts of *Bifidobacterium* (Figure 3; Pearson correlation: r = −0.41, n = 22, p<0.05) (but not *Paralactobacillus*; Pearson correlation: r = −0.27, n = 22, p = 0.232). Additionally, *Melissococcus*, the causative agent of European foul brood, was never detected in colonies with high loads of *Bifidobacterium* (mean 4.7% load across bee-gut samples) and was found only in colonies with a reduced load of *Bifidobacterium* species (mean 2.7%) which suggests that *Bifidobacterium*, many species of which are probiotic in other systems [77], may provide a measure of protection for honey bees against infection.

**Table 2:** Genetically diverse colonies are host to more active, potentially probiotic genera and fewer potentially pathogenic genera.

Genus	Description	Genetically uniform (24,580)	Genetically diverse (31,976)
*Serratia*	Entomopathogenic organism in *Drosophila* [121], [122]; insecticidal toxins [123]	95 (7/10)	65 (5/12)
*Brenneria*	Necrogenic plant pathogen; causative agent of deep bark canker [124]	585 (7/10)	282 (5/12)
*Klebsiella*	Opportunistic animal pathogen causing bacterial sepsis in gypsy moths [125]	300 (7/10)	117 (5/12)
*Melissococcus*	Bee pathogen, causative agent of European foul brood [126], [127]	79 (2/10)	1 (1/12)
*Bifidobacteria*	Probiotic organism associated with bees [47]	1,037 (10/10)	1,616 (12/12)
*Paralactobacillus*	Probiotic organism that may protect bees from pathogen infection [128]	2,479 (10/10)	3,307 (12/12)

Number of sequences in bee guts sampled from genetically uniform and genetically diverse colonies, classified into potential pathogenic and probiotic genera. Total number of sequences sampled for each colony type is given in parentheses in the header bar, and fraction of colonies sampled that had these pathogenic or probiotic genera is in parentheses for each genus. The same 7 genetically uniform and 5 genetically diverse colonies had these pathogens present (i.e., a colony either had all three pathogens in it, or none of them).

### Genetically diversity enhances the breadth and quality of active microbiotas in honey bee colonies

We sought to determine whether the diversity or composition of a colony's active microbiota was enhanced by increasing the genetic diversity of its worker population. A Mann-Whitney U test did not reveal a statistical difference between colony types in the number of active species (*U* = 63, p = 0.43), which is not unexpected given our relatively small number of focal colonies, so we employed a bootstrap analysis (5,000 runs that randomly resampled colonies from the genetically diverse and uniform treatment groups to generate a mean difference in the mean number of unique OTUs for each group) because it is commonly used to yield greater power for discriminating statistical differences when datasets have low numbers of replicates [78]. The bootstrap analysis revealed that colonies with genetically diverse worker populations showed significantly greater diversity of active bacterial species than colonies with genetically uniform worker populations [the 95% confidence interval (CI) for mean difference in species diversity between genetically diverse and uniform colonies exceeded 0; mean difference value and 95% CI for number of active species: 68, 64–73; Table 2, Table S2]. Across all colonies within each treatment group, 1,105 unique bacterial species (at 97% sequence identity) were found in genetically diverse colonies and 781 species were found in genetically uniform colonies. Furthermore, genetically diverse colonies had higher numbers of sequences affiliated to active bacteria of known beneficial genera and lower numbers of sequences affiliated to genera known to be harmful than colonies that were genetically uniform (mean difference values and 95% CIs for number sequences of known pathogens: 604, 587–614; for number of *Bifidobacterium* sequences: 388, 376–392; Table 2). The activity of known animal and plant pathogens in the digestive tract of workers based on number of sequences was 127% higher in genetically uniform colonies compared to those that were genetically diverse, and this load included the known bee pathogen *Melissococcus* (although no symptoms of disease were observed in any of our colonies; Table 2). Conversely, genetically diverse colonies had 40% greater activity of the beneficial probiotic genera *Bifidobacterium* and *Paralactobacillus* (Table 2).

## Discussion

Bacteria are remarkably abundant in honey bee colonies—it is estimated that the honey bee gut alone contains 10^8^ to 10^9^ bacteria per gram of contents [79]. Historically, most attempts to characterize these numerous microbes have taken a culture-based approach (reviewed by Gilliam 1997), which inserts inherent bias into the catalog of microbes that are subsequently identified in colonies. More recent studies have used culture-independent, 16S rRNA gene sequence analyses to probe this community based on single-strand conformation polymorphism [80], clone libraries [46]–[48], and a metagenomic project [4]. With only limited exceptions [4], other authors have focused on DNA that has been isolated from bee samples (which can include contaminating organisms that are not part of the active bacterial community [81]–[83]) and have been relatively small in scale, sampling anywhere from tens to a few hundred 16S rRNA sequences at most (compared to 70,562 sequences analyzed herein). Our study differs from these previous surveys of honey bee microbiotas because it is the first to use 454-pyrosequencing to deeply sample the *active* bacterial communities (i.e., those that are producing RNA) that are associated with several colony environments. Studies using 454-amplicon pyrosequencing show overwhelmingly that the most abundant and active microbes in an environment produce the largest amount of rRNA in those environments [84]–[87], so our methods allow us to comprehensively catalog active colony microbiomes without culture bias or confusion about species that are present but not functioning in colonies. We believe that this technique, in combination with a comprehensive sampling regimen within and across numerous colonies, accounts for the surprising discoveries we made about the composition of active microbiotas in honey bee colonies. Other explanations for the differences between our study and others may be the details of the molecular and computational techniques that were employed (e.g., primer pairs utilized, protocols for extracting nucleic acids, sequence processing, or databases queried [88]) or the age of bees that were sampled, which can produce shifts in microbiome composition as individuals mature (see [89], [90]). The influence of sampling geography is probably limited because there is strong empirical support for stable and species-specific gut microflora over large geographic areas [91], [92], including for subspecies of *A. mellifera* [4].

Importantly, the two dominant genera identified by our study (*Succinivibrio* and *Oenococcus*) have never been identified in honey bee colonies, which suggests that the major microbiotic players that are associated with honey bees were overlooked by previous methodologies. Indeed, the fact that known *Succinivibrio* isolates are obligate anaerobes may have precluded their identification by more conventional culturing attempts, which is a significant omission given that it made up a large fraction of the core group of active microbes that were found across all colony environments that we sampled. Additionally, gram-positive organisms such as *Oenococcus* would not have been identified by prior studies unless efforts were made to properly lyse these cells. Our comprehensive method for characterizing active honey bee microbiota allowed us to make three important and novel findings, including the discovery in honey bee colonies of genera associated with food processing and fermentative pathways in other habitats, the inverse relationship between the activity of known probiotic genera and known bacterial pathogens, and the observation that queen polyandry generates more diverse and, possibly, healthier microbiota within colonies. The strong association that honey bees have with microbes mirrors the heavy reliance of other social insects on bacterial symbionts to process relatively indigestible forage into nutritious food for their hosts [25] and to provide protection against pathogens [93]. The bacterial genera identified in this study suggest targets for investigating the means by which microbes can benefit social insect colonies, such as providing either a protective or a nutritional advantage to hosts by preventing colonization of pathogenic bacteria and/or by enhancing the bioavailability of nutrients in the foods that colony populations produce and consume.

We identified many microorganisms in honey bee colonies that are associated with fermentation in anaerobic habitats. Cultured *Succinivibrio* species are obligate anaerobes that cannot grow at atmospheric concentrations of oxygen, and we found that close relatives were active in significant numbers within bee guts. Organisms identified as *Succinivibrionaceae* across bee samples were 80–90% identical to known isolates. Characterized species from the *Succinivibrio* genus include *S. dextrinosolvens* and *S. amylolytica*, both of which have been isolated from cow rumens and are known to play an important role in the fermentation of starches and the production of large quantities of acetic and succinic acids in that environment [73], [94]. The starch-rich bee gut likely provides an ideal habitat for *Succinivibrionaceae*. Additionally, *Succinivibrio* could accomplish in this environment the anaerobic fermentation of the sugars that bees consume, a process that has been suspected by other authors to occur [37]. Similar composition of the plant polysaccharide-digesting communities in cow rumens and bee guts is a striking illustration of convergence in two different host-associated microbiomes. Metatranscriptomic sequencing of the bee gut would provide us with a more complete picture of the function that these *Succinivibrio*-like organisms serve within this microbial community. A need for an anaerobic environment may explain the lack of *Succinivibrionaceae* activity in bee bread; harvested pollen probably does not become anaerobic for quite some time (if at all) after it is inoculated with microbes and packed into cells by workers. However, it is possible that bee bread is stratified with respect to oxygen tension, which may allow it to support a range of bacteria, including aerobes and strict anaerobes, as community structure changes with food processing and maturation.

Bee guts and bee bread are known to be highly acidic environments [80] and could reflect or select for the presence of lactic acid bacteria (LABs). Important LABs that were identified in our study include *Oenococcus*, *Bifidobacterium*, and *Paralactobacillus*, all of which are facultative anaerobes. The activity of these LABs within both bee bread and bee guts suggests that these environments may be anaerobic at times. *Oenococcus* is obligately heterofermentative and well known for its participation in malolactic fermentation of wine (the decarboxylation of malate to produce lactate; [95]. *Oenococcus* utilizes hexoses and pentoses as carbohydrate sources, including cellobiose, the disaccharide component of cellulose [74]. It has been hypothesized that the osmotic potential of the bee gut may help to break open pollen grains and facilitate digestion [31], but our data suggest another possible means for breaking down tough pollen walls during bee-bread fermentation. Hemicellulose is easily hydrolyzed by acid, and the acidification of bee bread as it is fermented by LABs may help to break down complex plant carbohydrates into their constituent disaccharides, which could then be processed by other fermentative organisms within the community.

Indeed, other acid-tolerant bacteria, such as the acetic acid bacteria (AABs), have been suggested to play a large role in the bee-gut community based on their culturability as well as their presence in 16S rRNA clone libraries [96]. Only two species of AABs were found in our dataset: *Saccharibacter* and *Swaminathania* species. In total, AABs make up less than 3% of the total active honey bee microbiota (based on sequence abundance). Although microbes that exist in small numbers can certainly impact a community, either through secretion of metabolites or by seeding effects [97], the fact that we used cDNA as a template for our sequencing argues against a very active AAB community within honey bee colonies.

The presence of two potentially probiotic LABs in our samples deserves specific attention. *Bifidobacterium* and *Paralactobacillus* species are well known for their involvement in the fermentation of yogurt and other food products, respectively [98]. Both genera are within the Lactobacillaceae, the LAB group that includes important organisms that are involved in the production of Japanese sake (*Lactobacillus sakei*) and wine (*L. casei*) [75]. They are facultatively heterofermentative lactobacilli with the ability to use a range of hexoses and pentoses, including cellobiose [76]. *Bifidobacterium* has been found previously to associate with social insects [99], including honey bees [4], [48], [100], and one strain of *Paralactobacillus* has been patented for its ability to protect against pathogens [101]. We found that the more active the *Bifidobacterium* community was in bee guts, the lower was the activity of bacterial genera to which known pathogens belong. This correlative relationship suggests the possibility that *Bifidobacterium* may provide health benefits to bees, perhaps by modulating their immune response [102] or by excluding pathogens [103]. The particular *Bifidobacterium* organisms that were identified by this study may provide excellent probiotic activity for honey bees and we have targeted them for culture.

A central aspect of our study was to explore the effect that queen polyandry—and the genetic diversity that it introduces into colonies—has on colony microbiotas. Our analyses revealed that genetically diverse colonies had more diverse active microbiotas at the species level than genetically uniform colonies. This finding echoes the observation from ecological studies that genetic diversity within host populations begets diversity in other parts of the community that those populations support [104]–[106] including microbial communities [107]. Having a variety of host genotypes in the same colony, each of which may be associated with a different microbiota, is one means by which gut diversity could be increased in a colony with a polyandrous queen. Alternatively, each worker may share the same broader microbiome that is associated with her nestmates. There were also intriguing differences between colony types in microbiota content, specifically the number of sequences affiliating with bacterial genera whose members are known to be harmful or helpful. We observed consistently lower numbers of sequences from potentially pathogenic genera in genetically diverse colonies compared to genetically uniform colonies, a pattern that persisted across several genera (Table 2). Many of these genera have been found previously to associate with honey bee colonies [46], [47], [80], although their specific pathologies (if any for honey bees) are not all characterized. We also observed higher numbers of sequences that were affiliated with potentially probiotic genera in colonies with high levels of diversity relative to those with low diversity (Table 2). These key findings are aligned with observations that genetically diverse colonies have reduced expression of symptoms when infected with bacterial pathogens compared to colonies that lack such diversity in their work forces [56]–[58] although the role that beneficial microbes might play in modulating colony response is presently open to speculation. Microbial diversity in healthy, genetically diverse colonies may provide colonization resistance to pathogens [108], [109] and may be of extraordinary relevance to honey bee health, given that honey bees have a greatly reduced immune system relative to other model insects [110]. Our future research will seek to understand how intracolonial genetic diversity generates and maintains more diverse and healthful microbiotas and the selective advantage of this phenomenon for honey bee colonies. The need to uncover the potential that genetic diversity holds for improving colony health and productivity through enhanced nutrition is particularly urgent, given that poor nutrition is explicitly identified as a probable contributing factor in recent colony losses [111], [112] and persistent concerns about levels of genetic diversity in honey bee populations and the mating quality of queens in bee-breeding programs [12], [113].

## Materials and Methods

### Establishing bee colonies

Honey bee colonies (n = 30) were established for the study at Wellesley College (Wellesley, MA, USA) in 2010. To generate colony populations that were genetically diverse or uniform, the resident queen in each colony was removed and replaced with a queen that had been inseminated with 1 µL of semen that was harvested either from a group of 15 different drones or a single drone, rendering them multiply or singly mated, respectively (queens were reared at Glenn Apiaries, Fallbrook, CA). Inseminating drones were drawn at random from drone-bank colonies, each of which housed individuals from >10 source colonies derived from Carniolan, cordovan, hygienic Italian, and varroa sensitive hygienic lines kept by the queen breeder (no more than 4 drones were taken per bank). Semen from each drone was used to inseminate only one of the experimental queens. All inseminated queens were daughters of a singly inseminated Carniolan queen and were therefore highly related to one another (r = 0.75), whereas the drones were drawn from across a wide range of colonies and were presumed to be unrelated. Queens were introduced into colonies on May 11 and were given two months to completely replace the previous queens' workers with their own, during which time colonies were monitored weekly for general health and queen vitality. Before sampling began, eight colonies were removed from the study because their queens were poor egg layers, which left 10 genetically uniform colonies and 12 genetically diverse colonies for which bacterial communities could be assessed.

### Sampling from honey bee colonies

To deeply characterize the bacterial communities that are associated with bees and bee bread, we performed 16S rRNA tagged pyrosequencing. Our sampling strategy included taking 4–5 samples per colony (either entire digestive tracts from worker bees, whole worker bees, or bee bread) and pooling samples within each colony for 16S rRNA amplification. We made sure that sampled workers were the same age within and across colonies to reduce variability that may have resulted from age or behavioral caste differences. We age-matched workers by putting frames with pupating brood from each focal colony into a 34°C incubator, letting workers emerge as adults overnight from cells, marking the newly emerged individuals with paint on their thorax, and returning all marked workers and frames to their source colony. Paint-marked workers were subsequently collected from colonies when they were 12 days of age. Bee-bread samples were taken from frames that were placed in colonies when the inseminated queens were introduced; at that time, the frames were only foundational wax and all honeycomb and comb contents were produced by the focal genetically diverse or uniform worker populations during the intervening two months between queen introduction and colony sampling. Appropriate precautions were taken in the field during sampling to prevent cross-contamination between samples and colonies, including the use of gloves and sterile sampling equipment. Great care was taken to store samples appropriately upon collection. Whole-bee and bee-bread samples were flash frozen in liquid nitrogen in the field to ensure that the structure of the bacterial communities that were sampled represented *in situ* diversity. Bee guts (entire digestive tract, including the honey stomach) were collected from workers in the field by immobilizing marked individuals on ice, dissecting out their guts, placing them immediately into RNA later (Invitrogen, Carlsbad, CA, USA), and storing all gut samples at 4°C until further processing.

### RNA isolation and cDNA synthesis

We specifically isolated whole RNA from our samples, which allowed us to determine the *active* bacterial community in colonies and to avoid including in our analyses dormant spores that may have been collected by the bees along with pollen. Whole bee samples were ground in liquid nitrogen before proceeding with further extractions; all other samples were processed as collected. Each sample was subjected to bead beating at 4°C for 7 minutes to ensure lysis of gram-positive cells. MoBio (California, USA) kits appropriate for each sample type were used according to the manufacturer's instructions to isolate RNA from bee-bread samples (RNA Powersoil Total RNA kit) and whole-bee and bee-gut samples (Ultraclean Tissue and Cells RNA Isolation kit). All RNA extractions were subjected to DNAse treatment to remove this contaminating nucleic acid. For each sample type per colony, individual RNA extractions were pooled, but only after they were normalized by concentration to ensure that each individual contributed equally to the pooled sample. Thus, each colony was represented by a single, pooled bee-gut, bee-bread, and whole-bee sample. These pooled extractions were then subjected to reverse transcriptase reaction using random hexamers to produce cDNA (Thermo Scientific, MA, USA). Concentrations of cDNA in each sample were evaluated by spectrophotometry (Nanodrop 2000, Wilmington, DE, USA) and gel electrophoresis and then normalized for the PCR reactions that are described below.

### 16S rRNA gene amplification and pyrosequencing of barcoded amplicons

The microbiotas found in samples of bee bread, bee guts and whole bees were analyzed by massively parallel barcoded-pyrosequencing. A fragment of the 16S rRNA gene (∼330 bp), that spanned the V1 and V2 hypervariable regions, was PCR amplified from cDNA. The universal bacterial primers 27F [114] and 338RII [115] were modified by adding ligation adaptors and/or barcodes (i.e., sample-identification sequences) to the 5′- ends (see metadata on DDBJ for primers). PCR was performed using a high-fidelity polymerase (Phusion Hot Start, Finnzymes, Lafayette, CO, USA), 50°C annealing temperature, 1500 ng template in 50 µL volumes and 25 cycles in order to limit the effects of PCR bias and errors that are introduced by non-proofreading polymerases. Amplicons, purified and concentrated to 50 µL using the Qiagen PCR Cleanup kits (Qiagen, Valencia, CA, USA), quantified using a Nanodrop 2000 (Wilmington, DE, USA) and standardized to 100 ng/µL, were used as templates for emulsion PCR using the emPCR kit II (Roche, Branford, CT). DNA was sequenced using a GS-FLX pyrosequencer (Roche) and the GS-Titanium kit (Roche) by Beckman Coulter Genomics (Danvers, MA, USA) on GS FLX Titanium Pico Titre Plates following Roche standard protocols.

### Sequence processing and analyses

FASTA-formatted sequences and corresponding quality scores were extracted from the .sff data file generated by the pyrosequencer using the GS Amplicon software package (Roche, Branford, CT). All data pre-processing, analysis of operational taxonomic units (OTUs), phylotype analysis and hypothesis testing were performed using modules implemented in the Mothur software platform [116]. Pooled sequences were binned according to the colony from which they were derived using the unique barcodes on the primers (these were removed prior to downstream analyses). Primer regions were also removed from the sequences at this point. Sequence length and quality were evaluated for each read; sequences were culled if the length was <300 bp and >500 bp, the average SFF quality score was <30, they contained any ambiguous base calls, or did not match any of the primers or barcode colony identifiers. The data set was simplified by using the “unique.seqs” command to generate a non-redundant (unique) set of sequences. Unique sequences were aligned using the “align.seqs” command and an adaptation of the Bacterial SILVA SEED database as a template (available at: http://www.mothur.org/wiki/Alignment_database). To ensure that we were analyzing comparable regions of the 16S rRNA gene across all reads, sequences that started before the 2.5-percentile or ended after the 97.5-percentile in the alignment were filtered. Sequences were denoised using the “pre.cluster” command. This command applies a pseudo-single linkage algorithm with the goal of removing sequences that are likely due to pyrosequencing errors [117]. A total of 2,154 potentially chimeric sequences were detected and removed using the “chimera.slayer” command [118]. Aligned sequences were clustered into OTUs (defined by 97% similarity) using the average neighbour algorithm. Rarefaction curves were plotted for each sample and a weighted UniFrac dendrogram [119] was generated using the UniFrac module implemented in Mothur. The UniFrac algorithm assigned a distance between different microbial communities based on the composition of lineages that were found in each sample. Importantly, UniFrac takes into account the phylogenetic relatedness of lineages in each sample. All community diversity parameters (Shannon-Weaver, Chao1, and Simpson's) were calculated as described in the Mothur software manual. Sequences were taxonomically classified by the RDP-II Naive Bayesian Classifier [120] using a 60% confidence threshold. Sequences that could not be classified to at least the kingdom level were excluded from subsequent diversity analyses. Venn diagrams and heatmap figures were generated using custom Perl scripts. Pyrosequence data sets are available through the EBI/DDBJ Sequence Read Archive accession number DRA000526. Based on these procedures, we use the term “species” throughout to refer to operational taxonomic units (OTUs) at a 97% sequence-identity threshold.

### Statistical Analyses

Pearson correlations and Mann Whitney U-tests utilized the classification data generated through the Mothur pipeline (described above) and were run in the statistical package SPSS. Bootstrap analyses (5,000 runs per analysis) were also based on classification data and means, standard deviations from the mean differences, as confidence intervals were run for 5,000 replicates using an in-house perl script. The bootstrap analysis was performed such that a randomly selected 10 of the 12 genetically diverse colonies were compared to the 10 genetically uniform colonies. For each sampling, the difference between colony types in total number of species as well as number of sequences affiliating with known pathogens or *Bifidobacterium* were calculated. 95% confidence intervals (CI) around mean difference values were calculated and the null hypothesis that there was no effect of increased within-colony diversity was rejected if zero was not included in the CI.

## Supporting Information

Figure S1 **Rank abundance plots for each sampled environment within honey bee colonies.** For each environment, OTUs are ranked in terms of abundance in the dataset such that the most common OTUs are leftmost and OTUs are increasingly rare to the right of the ordinal axis. The bee-gut samples contained many more rare OTUs (singletons and doubletons) compared to the bee-bread and whole-bee samples, likely a consequence of the deeper sequence sampling of this environment.
(TIF)

Figure S2 **Unifrac clustering repeated with normalized libraries recapitulates results from entire dataset.** Weighted, species-based (97% identity) Unifrac clustering of sampled environments in each study colony using normalized libraries across each environment type. Colony environments sampled are colored as follows: bee-gut samples are in lavender; whole-bee samples are in green, and bee-bread samples are in pink, as in Figure 2.
(TIF)

Figure S3 **Comparisons between colony environments using normalized libraries.** Venn diagram representation of species-level diversity (97% identity) of the active bacterial communities that were found within three bee-associated sampling environments (bee bread, bee guts, and whole bees), pooled across colony type and normalized for library size. The total species richness in the dataset was 216 OTUs, with the most species-rich environment being bee guts (174 total species).
(TIF)

Table S1 **Anaerobic genera found associated with honey bee samples.** The number of unique sequences affiliating with facultative (F) and obligate (O) anaerobes found in all three bee-associated sampling environments (bee guts, bee bread, and whole bees).
(DOCX)

Table S2 **Sequencing statistics by sample.** All diversity metrics generated in the analysis of each colony sample. Columns are, in order: label (divergence level), group (colony identifier), nseqs (number of sequences, coverage, npshannon, simpson, simpson_lci, simpson_hci, sobs, chao, chao_lci, chao_hci,
(PDF)

Table S3 **Full classifications of sequences by sample.** Each of the taxonomic levels identified in each of the colony samples sorted by rankID (column 2). Columns are, in order: taxlevel, rankID, taxon, daughterlevels, total followed by a column for each colony sample.
(PDF)
