# Supplementary figures and images for: Characterization of the Active Microbiotas Associated with Honey Bees Reveals Healthier and Broader Communities when Colonies are Genetically Diverse
Source: PLoS One. 2012 Mar 12;7(3):e32962. doi: 10.1371/journal.pone.0032962 (PMC3299707; doi:10.1371/journal.pone.0032962)

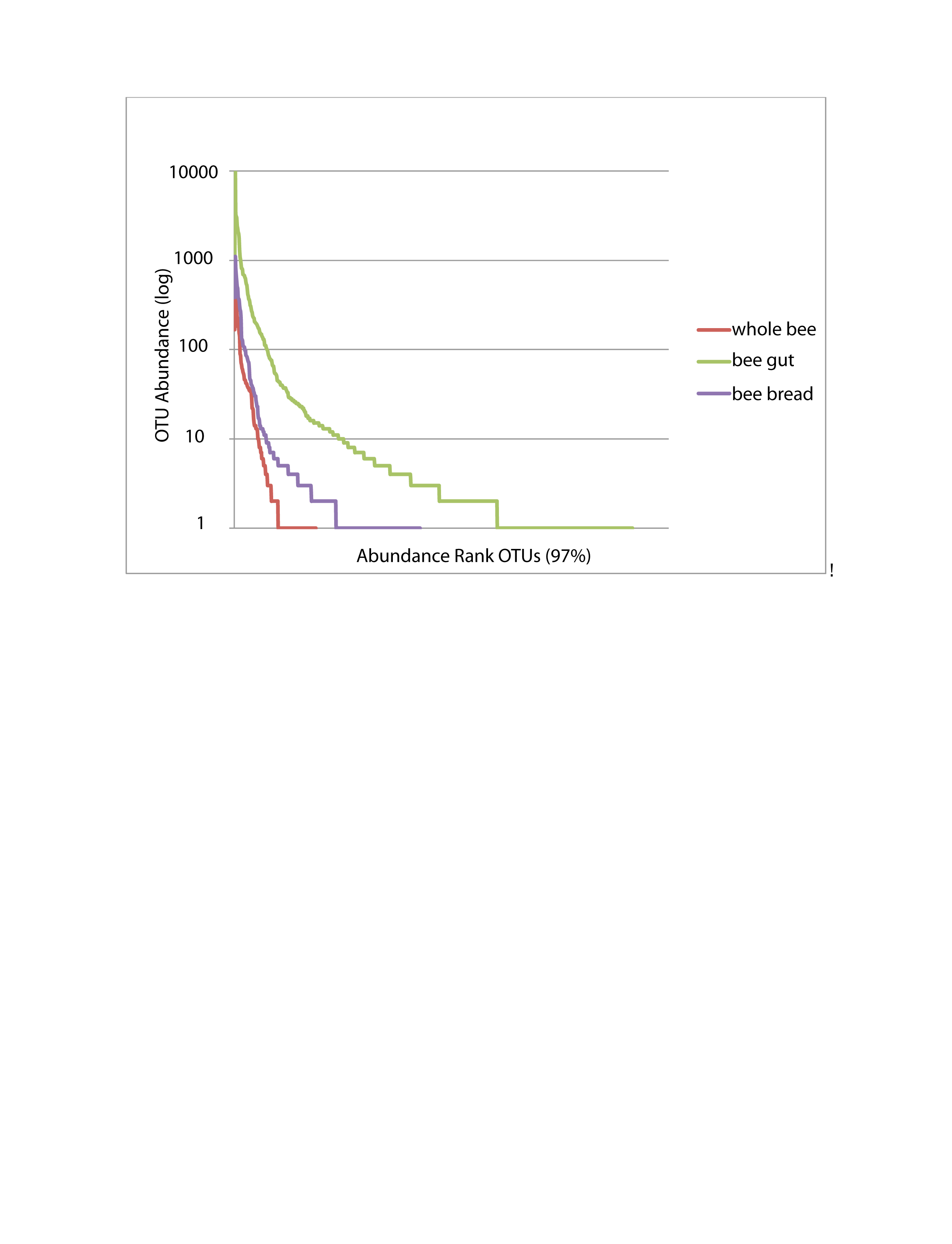

Supplement: Figure S1 — Rank abundance plots for each sampled environment within honey bee colonies. For each environment, OTUs are ranked in terms of abundance in the dataset such that the most common OTUs are leftmost and OTUs are increasingly rare to the right of the ordinal axis. The bee-gut samples contained many more rare OTUs (singletons and doubletons) compared to the bee-bread and whole-bee samples, likely a consequence of the deeper sequence sampling of this environment. (TIF) [file pone.0032962.s001.tif]

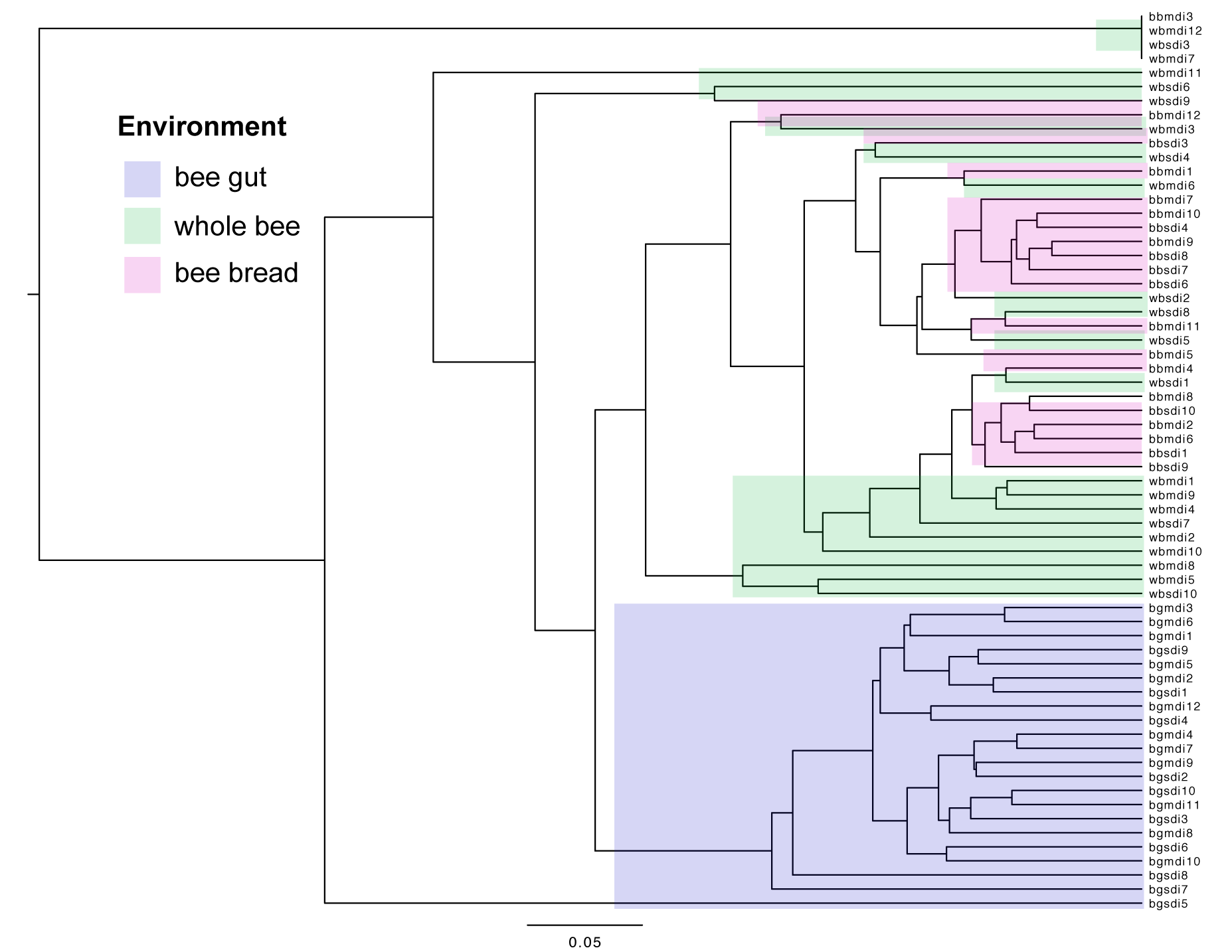

Supplement: Figure S2 — Unifrac clustering repeated with normalized libraries recapitulates results from entire dataset. Weighted, species-based (97% identity) Unifrac clustering of sampled environments in each study colony using normalized libraries across each environment type. Colony environments sampled are colored as follows: bee-gut samples are in lavender; whole-bee samples are in green, and bee-bread samples are in pink, as in Figure 2. (TIF) [file pone.0032962.s002.tif]

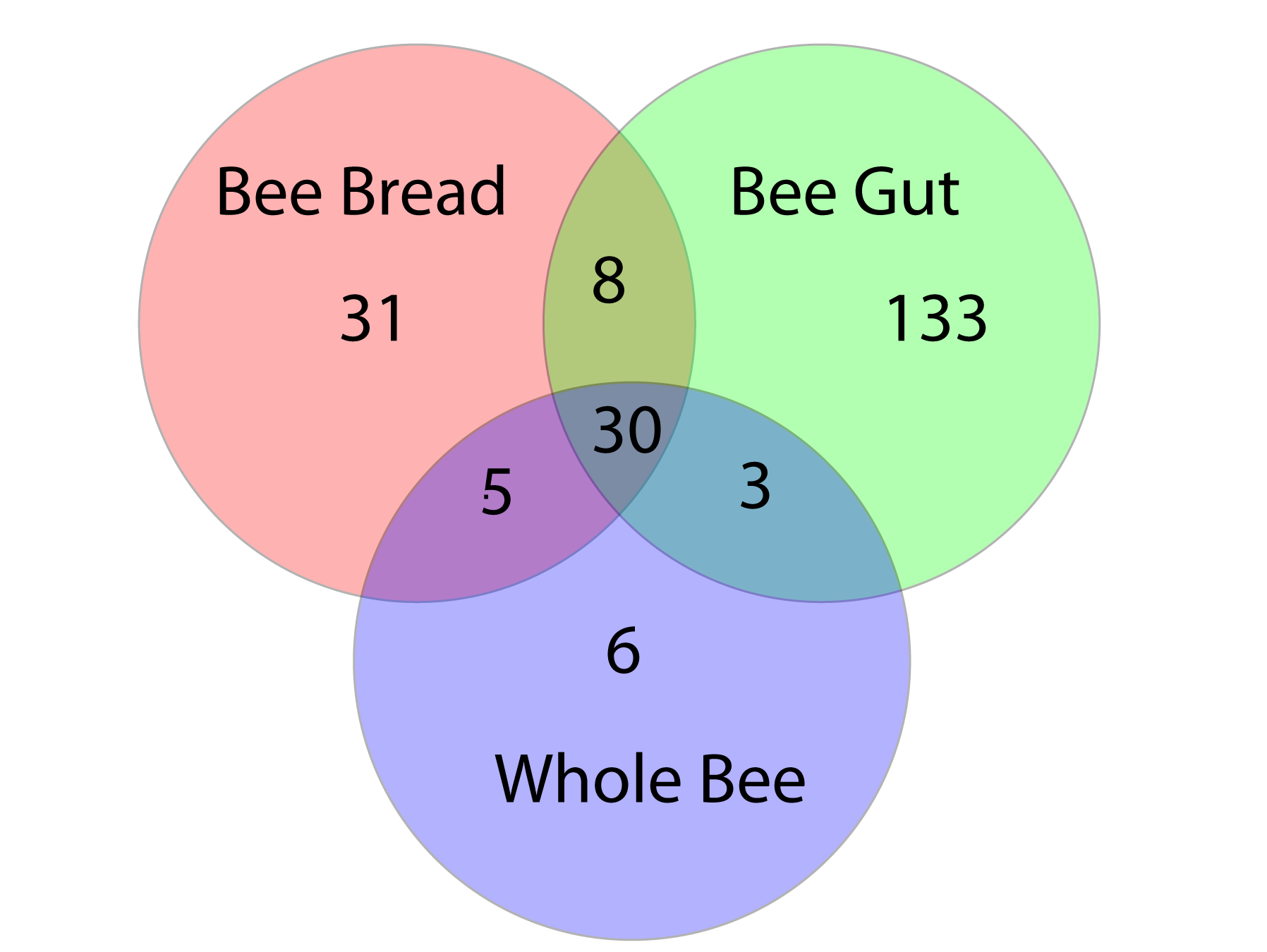

Supplement: Figure S3 — Comparisons between colony environments using normalized libraries. Venn diagram representation of species-level diversity (97% identity) of the active bacterial communities that were found within three bee-associated sampling environments (bee bread, bee guts, and whole bees), pooled across colony type and normalized for library size. The total species richness in the dataset was 216 OTUs, with the most species-rich environment being bee guts (174 total species). (TIF) [file pone.0032962.s003.tif]
