# Supplementary material for: Characterization of the Active Microbiotas Associated with Honey Bees Reveals Healthier and Broader Communities when Colonies are Genetically Diverse
Source: PLoS One. 2012 Mar 12;7(3):e32962. doi: 10.1371/journal.pone.0032962 (PMC3299707; doi:10.1371/journal.pone.0032962)
Supplement: Table S1 — Anaerobic genera found associated with honey bee samples. The number of unique sequences affiliating with facultative (F) and obligate (O) anaerobes found in all three bee-associated sampling environments (bee guts, bee bread, and whole bees). (DOCX) [file pone.0032962.s004.docx]

| Genus | Description | Total  (70 562) |
| --- | --- | --- |
| *Succinivibrio* | O | 21 961 |
| *Oenococcus* | F | 12 027 |
| *Paralactobacillus* | F | 9 218 |
| *Bowmanella* | F | 8 106 |
| *Bifidobacterium* | F | 2 811 |
| *Enterobacter* | F | 1 529 |
| *Laribacter* | F | 699 |
| *Klebsiella* | F | 601 |
| *Rummeliibacillus* | F | 590 |
| *Atopobacter* | F | 436 |
| *Escherichia/Shigella* | F | 286 |
| *Citrobacter* | F | 153 |
| *Pantoea* | F | 92 |
| *Lactobacillus* | F | 55 |
| *Kluyvera* | F | 23 |
| *Cronobacter* | F | 21 |
| *Pseudomonas* | F | 13 |
