# Supplementary material for: Characterization of the Active Microbiotas Associated with Honey Bees Reveals Healthier and Broader Communities when Colonies are Genetically Diverse
Source: PLoS One. 2012 Mar 12;7(3):e32962. doi: 10.1371/journal.pone.0032962 (PMC3299707; doi:10.1371/journal.pone.0032962)
Supplement: Table S2 — Sequencing statistics by sample. All diversity metrics generated in the analysis of each colony sample. Columns are, in order: label (divergence level), group (colony identifier), nseqs (number of sequences, coverage, npshannon, simpson, simpson_lci, simpson_hci, sobs, chao, chao_lci, chao_hci, (PDF) [file pone.0032962.s005.pdf]

label	group	nseqs	coverage	npshannon	simpson	simpson_lci	simpson_hci	sobs
0.03	bbmdi1	106	0.90566	3.141804	0.05858	0.042001	0.07516	28
0.03	bbmdi10	486	0.917695	3.707658	0.046566	0.039311	0.05382	82
0.03	bbmdi11	375	0.922667	3.514479	0.055358	0.046121	0.064596	65
0.03	bbmdi12	16	0.3125	3.661361	0.033333	-0.014842	0.081509	13
0.03	bbmdi2	469	0.921109	3.836149	0.037277	0.03203	0.042524	84
0.03	bbmdi3	5	0.4	2.28205	0.1	-0.070889	0.270889	4
0.03	bbmdi4	482	0.923237	3.744754	0.047705	0.039786	0.055624	84
0.03	bbmdi5	493	0.922921	3.778697	0.047082	0.039471	0.054693	88
0.03	bbmdi6	980	0.939796	3.841225	0.044166	0.039661	0.048672	127
0.03	bbmdi7	714	0.936975	3.680613	0.054522	0.04691	0.062133	99
0.03	bbmdi8	665	0.936842	3.756681	0.046549	0.040274	0.052824	97
0.03	bbmdi9	1124	0.957295	3.579975	0.055946	0.050284	0.061608	108
0.03	bbsdi1	745	0.938255	3.705325	0.053121	0.046537	0.059705	104
0.03	bbsdi10	504	0.882937	4.08491	0.037971	0.031191	0.044751	113
0.03	bbsdi3	68	0.808824	3.154525	0.057068	0.037075	0.07706	25
0.03	bbsdi4	484	0.911157	3.709983	0.046353	0.039747	0.052958	84
0.03	bbsdi6	476	0.901261	3.694594	0.044989	0.039031	0.050947	83
0.03	bbsdi7	729	0.938272	3.672307	0.048923	0.043464	0.054382	97
0.03	bbsdi8	315	0.879365	3.778326	0.042281	0.034599	0.049963	73
0.03	bbsdi9	299	0.892977	3.750571	0.03762	0.030949	0.044291	66
0.03	bgmdi1	2528	0.964003	4.025098	0.033992	0.032004	0.03598	202
0.03	bgmdi10	2566	0.980125	3.359237	0.074809	0.069453	0.080165	131
0.03	bgmdi11	5298	0.976029	3.884686	0.051688	0.04907	0.054306	295
0.03	bgmdi12	2383	0.963911	3.624367	0.06275	0.058583	0.066917	186
0.03	bgmdi2	2730	0.973626	3.252278	0.098898	0.091345	0.10645	147
0.03	bgmdi3	798	0.971178	3.040175	0.121279	0.10337	0.139187	67
0.03	bgmdi4	2550	0.96	4.065329	0.044272	0.04038	0.048164	229
0.03	bgmdi5	2316	0.973661	3.789067	0.051802	0.046799	0.056805	151
0.03	bgmdi6	2743	0.981772	3.358403	0.073778	0.068337	0.07922	126
0.03	bgmdi7	3043	0.975025	3.641896	0.060682	0.057043	0.064321	190
0.03	bgmdi8	2712	0.978614	3.163185	0.127471	0.117823	0.13712	149
0.03	bgmdi9	2309	0.961022	4.003748	0.037794	0.035111	0.040477	196
0.03	bgsdi1	2742	0.96353	3.970726	0.054476	0.050123	0.058829	234
0.03	bgsdi10	2923	0.970578	3.454574	0.096442	0.088614	0.104269	194

0.03	bgsdi2	2092	0.963671	3.940382	0.040883	0.037315	0.044451	172
0.03	bgsdi3	2682	0.972036	3.43556	0.106166	0.0971	0.115232	178
0.03	bgsdi4	2690	0.97658	3.234917	0.092623	0.087021	0.098225	159
0.03	bgsdi5	2156	0.982375	2.933647	0.136159	0.126047	0.146271	105
0.03	bgsdi6	2171	0.975127	3.295812	0.080996	0.074372	0.08762	118
0.03	bgsdi7	2831	0.979159	3.196403	0.088002	0.082715	0.09329	137
0.03	bgsdi8	2097	0.964711	3.670306	0.056267	0.052049	0.060484	161
0.03	bgsdi9	2196	0.959016	3.994829	0.046965	0.042278	0.051653	197
0.03	wbmdi1	433	0.95843	3.164544	0.069177	0.059458	0.078896	48
0.03	wbmdi10	31	0.451613	3.772646	0.032258	0.00386	0.060657	22
0.03	wbmdi11	8	0.75	1.578373	0.25	0.026101	0.473899	4
0.03	wbmdi12	2	0	0	0	0	0	2
0.03	wbmdi2	119	0.907563	2.923732	0.100271	0.067146	0.133396	28
0.03	wbmdi3	20	0.65	2.678194	0.084211	0.026694	0.141727	11
0.03	wbmdi4	159	0.874214	3.228288	0.077701	0.051629	0.103772	39
0.03	wbmdi5	80	0.8125	3.162735	0.075949	0.044356	0.107543	28
0.03	wbmdi6	190	0.936842	3.299104	0.054024	0.041903	0.066145	38
0.03	wbmdi7	6	0.333333	2.69414	0.066667	-0.058426	0.191759	5
0.03	wbmdi8	20	0.6	3.068997	0.047368	0.011149	0.083588	13
0.03	wbmdi9	1299	0.979215	3.096669	0.095951	0.085546	0.106357	74
0.03	wbsdi1	798	0.991228	2.681169	0.107178	0.097362	0.116995	36
0.03	wbsdi10	33	0.727273	3.062748	0.054924	0.024948	0.0849	17
0.03	wbsdi2	270	0.911111	3.439723	0.057056	0.045146	0.068967	53
0.03	wbsdi3	32	0.75	2.713603	0.078629	0.044591	0.112668	14
0.03	wbsdi4	116	0.896552	3.368241	0.042129	0.033041	0.051217	33
0.03	wbsdi5	353	0.915014	3.657738	0.048835	0.03868	0.058989	68
0.03	wbsdi6	18	0.444444	3.28884	0.052288	-0.008827	0.113402	13
0.03	wbsdi7	58	0.913793	2.782503	0.091349	0.052489	0.130209	18
0.03	wbsdi8	371	0.938005	3.366565	0.067079	0.054711	0.079448	58
0.03	wbsdi9	55	0.763636	3.059717	0.065993	0.039523	0.092463	22

chao	chao_lci	chao_hci
34.428571	29.513632	55.302896
160	115.43361	263.97257
110.111111	82.473811	181.460705
40.5	19.663101	126.498205
150.6	111.984962	242.497984
5.5	4.149231	19.077277
135.230769	106.115431	202.676942
127.055556	105.375515	175.786543
201.391304	165.062065	272.395846
160.875	127.723165	232.290171
147.647059	120.164297	207.736127
174.352941	139.491987	247.804226
149	125.494082	198.211978
203.052632	158.392301	291.653125
44.5	30.215203	97.911837
166.090909	120.241436	269.945096
218.125	141.100921	397.259486
155.235294	124.163287	221.850481
131.583333	98.292845	208.690822
128	90.37919	223.675457
322.441176	271.630534	410.329249
198.105263	163.640905	268.9593
517.25	431.783854	656.117639
321.37037	262.118444	426.745031
317.4	234.088996	480.407907
92.3	76.117964	137.200978
368.216216	311.721101	463.295176
252.666667	202.369041	352.212849
202.5625	162.521534	286.503017
304	252.155402	399.088827
206	178.388111	259.554912
344.333333	280.121429	457.559724
371.5	315.255904	466.6754
285.375	246.641899	352.607323

277.555556	229.890914	364.465011
289	238.353598	382.146901
211.783784	187.041363	258.357789
163.583333	130.292845	240.690822
207.4375	161.45489	302.077476
232.055556	184.675232	326.523117
269.04	219.578852	360.263748
345.333333	281.121429	458.559724
73.5	56.049017	128.786266
56	32.103432	136.416565
4.5	4.030262	12.261169
3	2.081128	14.326222
37.166667	30.29218	64.658448
21.5	13.03458	65.188088
102.333333	58.204468	247.863438
49	34.067775	100.679028
47.428571	40.47555	73.910389
8	5.39133	27.998494
20	14.451873	46.749509
124.142857	92.474901	210.093075
40.2	36.789764	58.335782
24.2	18.60153	49.369051
87.5	65.417223	148.854767
28	16.920488	81.112081
41.25	35.162057	64.480432
111.5	85.206802	177.971046
28	16.502488	77.24005
19.25	18.164244	27.513299
81	66.329788	121.507017
48	28.815969	121.178856
