# Supplementary material for: Characterization of the Active Microbiotas Associated with Honey Bees Reveals Healthier and Broader Communities when Colonies are Genetically Diverse
Source: PLoS One. 2012 Mar 12;7(3):e32962. doi: 10.1371/journal.pone.0032962 (PMC3299707; doi:10.1371/journal.pone.0032962)
Supplement: Table S3 — Full classifications of sequences by sample. Each of the taxonomic levels identified in each of the colony samples sorted by rankID (column 2). Columns are, in order: taxlevel, rankID, taxon, daughterlevels, total followed by a column for each colony sample. (PDF) [file pone.0032962.s006.pdf]

taxlevel	rankID	taxon	daughterlevels	total	bgsdi2	bgsdi4	bgmdi8	bgmdi10	bgmdi6
0	0	Root	1	0	0	0	0	0	0
1	0.1	Bacteria	8	70562	2092	2690	2712	2566	2743
2	0.1.1	Acidobacteria	1	3	0	0	0	0	0
2	0.1.2	Actinobacteria	1	2822	93	82	98	54	292
2	0.1.3	Aquificae	1	1	0	0	0	0	0
2	0.1.5	Bacteroidetes	3	21	1	0	0	0	0
2	0.1.11	Cyanobacteria	1	896	0	0	0	0	0
2	0.1.15	Firmicutes	2	26473	567	906	713	676	675
2	0.1.24	Proteobacteria	4	40342	1431	1702	1901	1836	1776
2	0.1.34	unclassified	1	4	0	0	0	0	0
3	0.1.1.1	Acidobacteria_Gp1	1	3	0	0	0	0	0
3	0.1.2.1	Actinobacteria	2	2822	93	82	98	54	292
3	0.1.3.1	Aquificae	1	1	0	0	0	0	0
3	0.1.5.2	Bacteroidia	1	2	0	0	0	0	0
3	0.1.5.3	Flavobacteria	1	18	1	0	0	0	0
3	0.1.5.4	Sphingobacteria	1	1	0	0	0	0	0
3	0.1.11.1	Cyanobacteria	1	896	0	0	0	0	0
3	0.1.15.1	Bacilli	3	26455	567	906	712	676	675
3	0.1.15.2	Clostridia	1	18	0	0	1	0	0
3	0.1.24.1	Alphaproteobacteria	4	1883	48	52	48	57	18
3	0.1.24.2	Betaproteobacteria	4	716	2	4	2	5	1
3	0.1.24.3	Deltaproteobacteria	2	13	0	0	0	0	0
3	0.1.24.5	Gammaproteobacteria	7	37730	1381	1646	1851	1774	1757
3	0.1.34.1	unclassified	1	4	0	0	0	0	0
4	0.1.1.1.1	Gp1	1	3	0	0	0	0	0
4	0.1.2.1.3	Actinomycetales	4	11	0	0	0	0	1
4	0.1.2.1.4	Bifidobacteriales	1	2811	93	82	98	54	291
4	0.1.3.1.1	Aquificales	1	1	0	0	0	0	0
4	0.1.5.2.1	Bacteroidales	1	2	0	0	0	0	0
4	0.1.5.3.1	Flavobacteriales	1	18	1	0	0	0	0
4	0.1.5.4.1	Sphingobacteriales	1	1	0	0	0	0	0
4	0.1.11.1.1	Chloroplast	1	896	0	0	0	0	0
4	0.1.15.1.1	Bacillales	7	4233	72	96	166	119	44
4	0.1.15.1.2	Lactobacillales	7	22184	495	808	546	556	631
4	0.1.15.1.3	unclassified	1	38	0	2	0	1	0
4	0.1.15.2.1	Clostridiales	3	18	0	0	1	0	0
4	0.1.24.1.2	Caulobacterales	1	2	0	0	0	0	0

4	0.1.24.1.5	Rhizobiales	4	154	6	4	0	0	0
4	0.1.24.1.7	Rhodospirillales	1	1725	42	48	47	57	18
4	0.1.24.1.10	Sphingomonadales	1	2	0	0	1	0	0
4	0.1.24.2.1	Burkholderiales	3	7	0	0	0	0	0
4	0.1.24.2.3	Methylophilales	1	7	0	0	0	1	0
4	0.1.24.2.4	Neisseriales	1	701	2	4	2	4	1
4	0.1.24.2.6	Procabacteriales	1	1	0	0	0	0	0
4	0.1.24.3.3	Desulfobacterales	1	6	0	0	0	0	0
4	0.1.24.3.6	Desulfuromonadales	1	7	0	0	0	0	0
4	0.1.24.5.2	Aeromonadales	2	22010	896	1304	1630	550	1412
4	0.1.24.5.3	Alteromonadales	3	11753	346	292	185	1224	321
4	0.1.24.5.6	Enterobacteriales	1	3878	139	49	35	0	24
4	0.1.24.5.11	Pasteurellales	1	2	0	0	0	0	0
4	0.1.24.5.12	Pseudomonadales	2	58	0	0	0	0	0
4	0.1.24.5.15	Xanthomonadales	1	3	0	0	0	0	0
4	0.1.24.5.16	unclassified	1	26	0	1	1	0	0
4	0.1.34.1.1	unclassified	1	4	0	0	0	0	0
5	0.1.1.1.1.1	unclassified	1	3	0	0	0	0	0
5	0.1.2.1.3.2	Actinomycetaceae	1	2	0	0	0	0	1
5	0.1.2.1.3.11	Corynebacteriaceae	1	1	0	0	0	0	0
5	0.1.2.1.3.29	Nocardioidaceae	1	1	0	0	0	0	0
5	0.1.2.1.3.32	Propionibacteriaceae	2	7	0	0	0	0	0
5	0.1.2.1.4.1	Bifidobacteriaceae	1	2811	93	82	98	54	291
5	0.1.3.1.1.2	Desulfurobacteriaceae	1	1	0	0	0	0	0
5	0.1.5.2.1.5	Prevotellaceae	1	2	0	0	0	0	0
5	0.1.5.3.1.2	Flavobacteriaceae	1	18	1	0	0	0	0
5	0.1.5.4.1.3	Cytophagaceae	1	1	0	0	0	0	0
5	0.1.11.1.1.5	Streptophyta	1	896	0	0	0	0	0
5	0.1.15.1.1.2	Bacillaceae	4	56	1	1	2	1	0
5	0.1.15.1.1.3	Bacillales_incertae_sedis	1	590	9	4	7	13	2
5	0.1.15.1.1.5	Listeriaceae	1	7	0	2	1	2	0
5	0.1.15.1.1.7	Planococcaceae	1	6	0	0	0	0	0
5	0.1.15.1.1.9	Staphylococcaceae	1	2	0	0	0	0	0
5	0.1.15.1.1.10	Thermoactinomycetaceae	1	3523	58	87	153	103	41
5	0.1.15.1.1.11	unclassified	1	49	4	2	3	0	1
5	0.1.15.1.2.2	Carnobacteriaceae	1	1	0	0	0	0	0
5	0.1.15.1.2.3	Enterococcaceae	2	520	13	6	13	1	2
5	0.1.15.1.2.4	Lactobacillaceae	2	9473	220	177	285	226	289

5	0.1.15.1.2.5	Leuconostocaceae	5	12081	258	625	244	329	339
5	0.1.15.1.2.6	Streptococcaceae	1	8	0	0	0	0	0
5	0.1.15.1.2.7	unclassified	1	91	2	0	3	0	1
5	0.1.15.1.2.8	unclassified_Lactobacillale	1	10	2	0	1	0	0
5	0.1.15.1.3.1	unclassified	1	38	0	2	0	1	0
5	0.1.15.2.1.1	Clostridiaceae	2	10	0	0	1	0	0
5	0.1.15.2.1.3	Gracilibacteraceae	1	7	0	0	0	0	0
5	0.1.15.2.1.5	Incertae_Sedis_XI	1	1	0	0	0	0	0
5	0.1.24.1.2.2	Hyphomonadaceae	1	2	0	0	0	0	0
5	0.1.24.1.5.1	Aurantimonadaceae	1	151	6	4	0	0	0
5	0.1.24.1.5.4	Bradyrhizobiaceae	1	1	0	0	0	0	0
5	0.1.24.1.5.7	Methylobacteriaceae	1	1	0	0	0	0	0
5	0.1.24.1.5.11	Rhodobiaceae	1	1	0	0	0	0	0
5	0.1.24.1.7.1	Acetobacteraceae	2	1725	42	48	47	57	18
5	0.1.24.1.10.2	Sphingomonadaceae	1	2	0	0	1	0	0
5	0.1.24.2.1.2	Burkholderiaceae	1	1	0	0	0	0	0
5	0.1.24.2.1.4	Comamonadaceae	3	4	0	0	0	0	0
5	0.1.24.2.1.5	Oxalobacteraceae	1	2	0	0	0	0	0
5	0.1.24.2.3.1	Methylophilaceae	1	7	0	0	0	1	0
5	0.1.24.2.4.1	Neisseriaceae	3	701	2	4	2	4	1
5	0.1.24.2.6.1	Procabacteriaceae	1	1	0	0	0	0	0
5	0.1.24.3.3.1	Desulfobacteraceae	1	6	0	0	0	0	0
5	0.1.24.3.6.2	Geobacteraceae	1	7	0	0	0	0	0
5	0.1.24.5.2.1	Aeromonadaceae	2	49	4	2	1	0	0
5	0.1.24.5.2.2	Succinivibrionaceae	1	21961	892	1302	1629	550	1412
5	0.1.24.5.3.1	Alteromonadaceae	1	8106	286	240	160	883	242
5	0.1.24.5.3.3	Colwelliaceae	1	3632	60	52	23	340	79
5	0.1.24.5.3.10	unclassified	1	15	0	0	2	1	0
5	0.1.24.5.6.1	Enterobacteriaceae	23	3878	139	49	35	0	24
5	0.1.24.5.11.1	Pasteurellaceae	1	2	0	0	0	0	0
5	0.1.24.5.12.1	Moraxellaceae	1	45	0	0	0	0	0
5	0.1.24.5.12.2	Pseudomonadaceae	1	13	0	0	0	0	0
5	0.1.24.5.15.2	Xanthomonadaceae	3	3	0	0	0	0	0
5	0.1.24.5.16.1	unclassified	1	26	0	1	1	0	0
5	0.1.34.1.1.1	unclassified	1	4	0	0	0	0	0
6	0.1.1.1.1.1.1	unclassified	0	3	0	0	0	0	0
6	0.1.2.1.3.2.2	Actinomyces	0	2	0	0	0	0	1
6	0.1.2.1.3.11.1	Corynebacterium	0	1	0	0	0	0	0

6	0.1.2.1.3.29.3	Marmoricola	0	1	0	0	0	0	0
6	0.1.2.1.3.32.1	Aestuariimicrobium	0	1	0	0	0	0	0
6	0.1.2.1.3.32.6	Propionibacterium	0	6	0	0	0	0	0
6	0.1.2.1.4.1.3	Bifidobacterium	0	2811	93	82	98	54	291
6	0.1.3.1.1.2.4	unclassified_Desulfurobac	0	1	0	0	0	0	0
6	0.1.5.2.1.5.4	unclassified_Prevotellacea	0	2	0	0	0	0	0
6	0.1.5.3.1.2.12	Empedobacter	0	18	1	0	0	0	0
6	0.1.5.4.1.3.6	Hymenobacter	0	1	0	0	0	0	0
6	0.1.11.1.1.5.1	unclassified	0	896	0	0	0	0	0
6	0.1.15.1.1.2.3	Bacillus	0	1	0	0	0	0	0
6	0.1.15.1.1.2.16	Salimicrobium	0	28	1	0	1	1	0
6	0.1.15.1.1.2.17	Salirhabdus	0	21	0	1	0	0	0
6	0.1.15.1.1.2.24	Vulcanibacillus	0	6	0	0	1	0	0
6	0.1.15.1.1.3.2	Rummeliibacillus	0	590	9	4	7	13	2
6	0.1.15.1.1.5.1	Brochothrix	0	7	0	2	1	2	0
6	0.1.15.1.1.7.2	Filibacter	0	6	0	0	0	0	0
6	0.1.15.1.1.9.5	Staphylococcus	0	2	0	0	0	0	0
6	0.1.15.1.1.10.5	Shimazuella	0	3523	58	87	153	103	41
6	0.1.15.1.1.11.1	unclassified	0	49	4	2	3	0	1
6	0.1.15.1.2.2.7	Granulicatella	0	1	0	0	0	0	0
6	0.1.15.1.2.3.1	Atopobacter	0	436	13	6	13	1	2
6	0.1.15.1.2.3.3	Melissococcus	0	84	0	0	0	0	0
6	0.1.15.1.2.4.1	Lactobacillus	0	55	0	0	0	4	0
6	0.1.15.1.2.4.2	Paralactobacillus	0	9418	220	177	285	222	289
6	0.1.15.1.2.5.1	Fructobacillus	0	19	0	0	0	0	0
6	0.1.15.1.2.5.2	Leuconostoc	0	1	0	0	0	0	0
6	0.1.15.1.2.5.3	Oenococcus	0	12027	257	625	244	329	339
6	0.1.15.1.2.5.4	Weissella	0	1	0	0	0	0	0
6	0.1.15.1.2.5.5	unclassified	0	33	1	0	0	0	0
6	0.1.15.1.2.6.2	Streptococcus	0	8	0	0	0	0	0
6	0.1.15.1.2.7.1	unclassified	0	91	2	0	3	0	1
6	0.1.15.1.2.8.1	unclassified	0	10	2	0	1	0	0
6	0.1.15.1.3.1.1	unclassified	0	38	0	2	0	1	0
6	0.1.15.2.1.1.5	Geosporobacter	0	7	0	0	0	0	0
6	0.1.15.2.1.1.6	Natronincola	0	3	0	0	1	0	0
6	0.1.15.2.1.3.1	Gracilibacter	0	7	0	0	0	0	0
6	0.1.15.2.1.5.1	Anaerococcus	0	1	0	0	0	0	0
6	0.1.24.1.2.2.2	Hirschia	0	2	0	0	0	0	0

6	0.1.24.1.5.1.1	Aurantimonas	0	151	6	4	0	0	0
6	0.1.24.1.5.4.1	Afipia	0	1	0	0	0	0	0
6	0.1.24.1.5.7.1	Meganema	0	1	0	0	0	0	0
6	0.1.24.1.5.11.1	Afifella	0	1	0	0	0	0	0
6	0.1.24.1.7.1.13	Saccharibacter	0	1693	42	45	47	57	18
6	0.1.24.1.7.1.15	Swaminathania	0	32	0	3	0	0	0
6	0.1.24.1.10.2.4	Sphingomonas	0	2	0	0	1	0	0
6	0.1.24.2.1.2.8	Ralstonia	0	1	0	0	0	0	0
6	0.1.24.2.1.4.8	Delftia	0	1	0	0	0	0	0
6	0.1.24.2.1.4.9	Diaphorobacter	0	1	0	0	0	0	0
6	0.1.24.2.1.4.16	Pelomonas	0	2	0	0	0	0	0
6	0.1.24.2.1.5.3	Herbaspirillum	0	2	0	0	0	0	0
6	0.1.24.2.3.1.1	Methylobacillus	0	7	0	0	0	1	0
6	0.1.24.2.4.1.11	Laribacter	0	699	2	4	2	4	1
6	0.1.24.2.4.1.13	Neisseria	0	1	0	0	0	0	0
6	0.1.24.2.4.1.16	Stenoxybacter	0	1	0	0	0	0	0
6	0.1.24.2.6.1.1	Procabacter	0	1	0	0	0	0	0
6	0.1.24.3.3.1.2	Desulfatiferula	0	6	0	0	0	0	0
6	0.1.24.3.6.2.3	Geopsychrobacter	0	7	0	0	0	0	0
6	0.1.24.5.2.1.1	Aeromonas	0	1	0	0	0	0	0
6	0.1.24.5.2.1.3	Tolumonas	0	48	4	2	1	0	0
6	0.1.24.5.2.2.2	Succinivibrio	0	21961	892	1302	1629	550	1412
6	0.1.24.5.3.1.4	Bowmanella	0	8106	286	240	160	883	242
6	0.1.24.5.3.3.4	unclassified_Colwelliaceae	0	3632	60	52	23	340	79
6	0.1.24.5.3.10.1	unclassified	0	15	0	0	2	1	0
6	0.1.24.5.6.1.1	Arsenophonus	0	5	0	0	0	0	0
6	0.1.24.5.6.1.2	Brenneria	0	870	0	5	0	0	1
6	0.1.24.5.6.1.3	Buttiauxella	0	9	1	0	0	0	0
6	0.1.24.5.6.1.4	Citrobacter	0	153	0	0	0	0	3
6	0.1.24.5.6.1.5	Cronobacter	0	21	0	0	0	0	0
6	0.1.24.5.6.1.6	Dickeya	0	5	0	0	0	0	0
6	0.1.24.5.6.1.8	Enterobacter	0	1099	66	30	3	0	10
6	0.1.24.5.6.1.9	Erwinia	0	1	1	0	0	0	0
6	0.1.24.5.6.1.10	Escherichia/Shigella	0	286	4	0	30	0	5
6	0.1.24.5.6.1.11	Klebsiella	0	601	48	0	2	0	0
6	0.1.24.5.6.1.12	Kluyvera	0	23	0	0	0	0	0
6	0.1.24.5.6.1.13	Morganella	0	1	0	0	0	0	0
6	0.1.24.5.6.1.14	Pantoea	0	92	1	0	0	0	5

6	0.1.24.5.6.1.15	Pectobacterium	0	9	0	0	0	0	0
6	0.1.24.5.6.1.19	Providencia	0	4	0	3	0	0	0
6	0.1.24.5.6.1.20	Rahnella	0	2	0	0	0	0	0
6	0.1.24.5.6.1.21	Raoultella	0	22	0	0	0	0	0
6	0.1.24.5.6.1.22	Salmonella	0	1	0	0	0	0	0
6	0.1.24.5.6.1.23	Samsonia	0	9	1	0	0	0	0
6	0.1.24.5.6.1.24	Serratia	0	198	15	10	0	0	0
6	0.1.24.5.6.1.28	Yersinia	0	5	0	0	0	0	0
6	0.1.24.5.6.1.29	unclassified	0	32	2	1	0	0	0
6	0.1.24.5.6.1.30	unclassified_Enterobacteri	0	430	0	0	0	0	0
6	0.1.24.5.11.1.9	Phocoenobacter	0	2	0	0	0	0	0
6	0.1.24.5.12.1.1	Acinetobacter	0	45	0	0	0	0	0
6	0.1.24.5.12.2.3	Pseudomonas	0	13	0	0	0	0	0
6	0.1.24.5.15.2.4	Dyella	0	1	0	0	0	0	0
6	0.1.24.5.15.2.1	Stenotrophomonas	0	1	0	0	0	0	0
6	0.1.24.5.15.2.1	Xanthomonas	0	1	0	0	0	0	0
6	0.1.24.5.16.1.1	unclassified	0	26	0	1	1	0	0
6	0.1.34.1.1.1.1	unclassified	0	4	0	0	0	0	0

bgmdi7	bgmdi9	bgsdi3	bgmdi12	bgmdi11	bgsdi7	bgsdi10	bgsdi1	bgmdi1	bgsdi5
0	0	0	0	0	0	0	0	0	0
3043	2309	2682	2383	5298	2831	2923	2742	2528	2156
0	0	2	0	0	0	0	0	0	0
90	95	111	54	122	35	61	254	207	171
0	0	0	0	0	0	0	0	0	0
0	1	2	0	0	0	0	0	2	0
0	0	0	0	0	0	0	0	0	0
848	645	689	825	1433	1005	961	937	804	320
2105	1567	1878	1504	3743	1791	1901	1551	1515	1665
0	1	0	0	0	0	0	0	0	0
0	0	2	0	0	0	0	0	0	0
90	95	111	54	122	35	61	254	207	171
0	0	0	0	0	0	0	0	0	0
0	0	0	0	0	0	0	0	2	0
0	1	2	0	0	0	0	0	0	0
0	0	0	0	0	0	0	0	0	0
0	0	0	0	0	0	0	0	0	0
846	644	689	825	1432	1005	961	937	804	320
2	1	0	0	1	0	0	0	0	0
9	61	21	6	42	6	18	35	36	28
20	12	2	10	3	439	0	5	4	0
0	1	0	0	0	0	4	2	1	0
2076	1493	1855	1488	3698	1346	1879	1509	1474	1637
0	1	0	0	0	0	0	0	0	0
0	0	2	0	0	0	0	0	0	0
0	0	0	0	0	0	0	0	0	0
90	95	111	54	122	35	61	254	207	171
0	0	0	0	0	0	0	0	0	0
0	0	0	0	0	0	0	0	2	0
0	1	2	0	0	0	0	0	0	0
0	0	0	0	0	0	0	0	0	0
0	0	0	0	0	0	0	0	0	0
91	118	124	99	172	89	136	208	72	34
753	523	565	721	1259	916	825	726	731	286
2	3	0	5	1	0	0	3	1	0
2	1	0	0	1	0	0	0	0	0
0	1	0	0	0	0	0	0	0	0

2	28	2	0	17	0	0	3	12	1
7	32	19	6	25	6	18	32	24	27
0	0	0	0	0	0	0	0	0	0
0	0	0	0	0	0	0	0	0	0
0	0	0	2	0	0	0	2	0	0
20	12	2	8	3	439	0	3	4	0
0	0	0	0	0	0	0	0	0	0
0	0	0	0	0	0	2	2	0	0
0	1	0	0	0	0	2	0	1	0
1021	978	1299	702	1665	854	1248	878	774	1439
1000	488	502	775	1672	147	614	583	687	192
53	27	54	9	353	343	16	47	12	5
0	0	0	0	1	0	0	0	0	0
0	0	0	0	0	0	0	0	0	0
1	0	0	0	0	0	0	0	0	0
1	0	0	2	7	2	1	1	1	1
0	1	0	0	0	0	0	0	0	0
0	0	2	0	0	0	0	0	0	0
0	0	0	0	0	0	0	0	0	0
0	0	0	0	0	0	0	0	0	0
0	0	0	0	0	0	0	0	0	0
0	0	0	0	0	0	0	0	0	0
90	95	111	54	122	35	61	254	207	171
0	0	0	0	0	0	0	0	0	0
0	0	0	0	0	0	0	0	2	0
0	1	2	0	0	0	0	0	0	0
0	0	0	0	0	0	0	0	0	0
0	0	0	0	0	0	0	0	0	0
4	1	2	6	8	0	1	1	2	0
1	13	5	3	19	6	63	27	38	0
0	0	0	0	0	0	0	0	0	0
0	0	0	0	0	0	1	1	0	0
0	0	0	0	0	0	0	0	0	0
84	99	114	86	141	82	70	172	29	34
2	5	3	4	4	1	1	7	3	0
0	0	0	0	0	0	0	0	0	0
18	6	0	10	50	76	6	2	6	0
292	167	270	234	470	382	396	298	470	80

441	348	289	475	730	456	419	413	248	206
0	0	0	0	0	0	0	0	0	0
1	2	6	2	9	2	4	13	7	0
1	0	0	0	0	0	0	0	0	0
2	3	0	5	1	0	0	3	1	0
1	1	0	0	0	0	0	0	0	0
1	0	0	0	0	0	0	0	0	0
0	0	0	0	1	0	0	0	0	0
0	1	0	0	0	0	0	0	0	0
2	28	2	0	16	0	0	3	12	1
0	0	0	0	0	0	0	0	0	0
0	0	0	0	0	0	0	0	0	0
0	0	0	0	1	0	0	0	0	0
7	32	19	6	25	6	18	32	24	27
0	0	0	0	0	0	0	0	0	0
0	0	0	0	0	0	0	0	0	0
0	0	0	0	0	0	0	0	0	0
0	0	0	0	0	0	0	0	0	0
0	0	0	2	0	0	0	2	0	0
20	12	2	8	3	439	0	3	4	0
0	0	0	0	0	0	0	0	0	0
0	0	0	0	0	0	2	2	0	0
0	1	0	0	0	0	2	0	1	0
2	1	1	4	4	1	3	1	2	1
1019	977	1298	698	1661	853	1245	877	772	1438
687	285	228	528	1029	23	472	189	634	106
313	201	273	247	643	124	142	392	53	86
0	2	1	0	0	0	0	2	0	0
53	27	54	9	353	343	16	47	12	5
0	0	0	0	1	0	0	0	0	0
0	0	0	0	0	0	0	0	0	0
0	0	0	0	0	0	0	0	0	0
1	0	0	0	0	0	0	0	0	0
1	0	0	2	7	2	1	1	1	1
0	1	0	0	0	0	0	0	0	0
0	0	2	0	0	0	0	0	0	0
0	0	0	0	0	0	0	0	0	0
0	0	0	0	0	0	0	0	0	0

0	0	0	0	0	0	0	0	0	0
0	0	0	0	0	0	0	0	0	0
0	0	0	0	0	0	0	0	0	0
90	95	111	54	122	35	61	254	207	171
0	0	0	0	0	0	0	0	0	0
0	0	0	0	0	0	0	0	2	0
0	1	2	0	0	0	0	0	0	0
0	0	0	0	0	0	0	0	0	0
0	0	0	0	0	0	0	0	0	0
0	0	0	0	0	0	0	0	0	0
0	0	0	1	2	0	0	1	1	0
4	1	1	3	4	0	1	0	1	0
0	0	1	2	2	0	0	0	0	0
1	13	5	3	19	6	63	27	38	0
0	0	0	0	0	0	0	0	0	0
0	0	0	0	0	0	1	1	0	0
0	0	0	0	0	0	0	0	0	0
84	99	114	86	141	82	70	172	29	34
2	5	3	4	4	1	1	7	3	0
0	0	0	0	0	0	0	0	0	0
18	6	0	10	50	1	6	2	6	0
0	0	0	0	0	75	0	0	0	0
0	0	0	0	0	1	0	2	1	0
292	167	270	234	470	381	396	296	469	80
0	0	2	0	0	0	0	1	1	0
0	0	0	0	1	0	0	0	0	0
441	348	285	474	727	456	417	412	247	206
0	0	0	0	0	0	0	0	0	0
0	0	2	1	2	0	2	0	0	0
0	0	0	0	0	0	0	0	0	0
1	2	6	2	9	2	4	13	7	0
1	0	0	0	0	0	0	0	0	0
2	3	0	5	1	0	0	3	1	0
0	0	0	0	0	0	0	0	0	0
1	1	0	0	0	0	0	0	0	0
1	0	0	0	0	0	0	0	0	0
0	0	0	0	1	0	0	0	0	0
0	1	0	0	0	0	0	0	0	0

2	28	2	0	16	0	0	3	12	1
0	0	0	0	0	0	0	0	0	0
0	0	0	0	0	0	0	0	0	0
0	0	0	0	1	0	0	0	0	0
6	31	19	6	25	6	16	32	22	27
1	1	0	0	0	0	2	0	2	0
0	0	0	0	0	0	0	0	0	0
0	0	0	0	0	0	0	0	0	0
0	0	0	0	0	0	0	0	0	0
0	0	0	0	0	0	0	0	0	0
0	0	0	0	0	0	0	0	0	0
0	0	0	0	0	0	0	0	0	0
0	0	0	2	0	0	0	2	0	0
20	12	2	8	3	439	0	3	4	0
0	0	0	0	0	0	0	0	0	0
0	0	0	0	0	0	0	0	0	0
0	0	0	0	0	0	0	0	0	0
0	0	0	0	0	0	2	2	0	0
0	1	0	0	0	0	2	0	1	0
0	0	0	0	0	0	0	0	0	0
2	1	1	4	4	1	3	1	2	1
1019	977	1298	698	1661	853	1245	877	772	1438
687	285	228	528	1029	23	472	189	634	106
313	201	273	247	643	124	142	392	53	86
0	2	1	0	0	0	0	2	0	0
0	0	0	0	1	0	0	0	0	0
0	0	2	5	244	319	0	12	0	0
0	2	0	0	0	0	0	0	0	0
0	0	0	0	1	0	0	0	0	0
0	0	0	0	0	1	1	0	0	0
0	0	0	0	0	0	0	0	3	0
26	16	35	0	24	11	6	21	0	0
0	0	0	0	0	0	0	0	0	0
18	2	1	2	13	0	0	0	2	1
0	1	4	0	12	12	1	1	0	0
0	0	0	0	1	0	1	1	0	0
0	0	0	0	0	0	0	0	0	0
8	1	1	1	3	0	2	0	0	0

0	0	1	0	0	0	1	1	0	0
0	0	0	0	1	0	0	0	0	0
0	0	0	0	0	0	0	0	0	0
0	0	0	0	1	0	0	0	0	0
0	0	0	0	0	0	0	0	0	0
0	0	0	0	1	0	0	0	0	0
0	5	7	0	48	0	4	11	7	4
1	0	2	0	0	0	0	0	0	0
0	0	1	1	3	0	0	0	0	0
0	0	0	0	0	0	0	0	0	0
0	0	0	0	1	0	0	0	0	0
0	0	0	0	0	0	0	0	0	0
0	0	0	0	0	0	0	0	0	0
0	0	0	0	0	0	0	0	0	0
0	0	0	0	0	0	0	0	0	0
1	0	0	0	0	0	0	0	0	0
1	0	0	2	7	2	1	1	1	1
0	1	0	0	0	0	0	0	0	0

bgmdi5	bgsdi6	bgmdi2	bgsdi9	bgsdi8	bgmdi4	bgmdi3	bbsdi4	bbsdi10	wbsdi1
0	0	0	0	0	0	0	0	0	0
2316	2171	2730	2196	2097	2550	798	484	504	798
0	0	0	1	0	0	0	0	0	0
136	89	251	109	32	139	80	1	4	23
0	0	0	0	0	0	0	0	0	0
0	0	1	0	0	1	0	0	1	0
0	0	0	0	0	0	0	30	36	0
639	637	972	670	519	672	202	327	372	695
1541	1445	1506	1416	1546	1738	516	126	91	80
0	0	0	0	0	0	0	0	0	0
0	0	0	1	0	0	0	0	0	0
136	89	251	109	32	139	80	1	4	23
0	0	0	0	0	0	0	0	0	0
0	0	0	0	0	0	0	0	0	0
0	0	0	0	0	1	0	0	1	0
0	0	1	0	0	0	0	0	0	0
0	0	0	0	0	0	0	30	36	0
632	637	972	670	519	671	202	327	369	695
7	0	0	0	0	1	0	0	3	0
8	35	20	88	5	42	0	57	24	62
55	6	4	6	1	13	0	3	9	1
0	0	2	1	0	1	0	0	1	0
1478	1404	1480	1321	1540	1682	516	66	57	17
0	0	0	0	0	0	0	0	0	0
0	0	0	1	0	0	0	0	0	0
0	0	1	0	0	0	0	0	0	0
136	89	250	109	32	139	80	1	4	23
0	0	0	0	0	0	0	0	0	0
0	0	0	0	0	0	0	0	0	0
0	0	0	0	0	1	0	0	1	0
0	0	1	0	0	0	0	0	0	0
0	0	0	0	0	0	0	30	36	0
62	33	168	79	70	129	13	70	88	175
570	603	804	588	449	539	189	257	278	520
0	1	0	3	0	3	0	0	3	0
7	0	0	0	0	1	0	0	3	0
0	0	0	0	0	0	0	0	0	0

0	4	1	42	0	26	0	0	0	0
8	30	19	46	5	16	0	57	24	62
0	1	0	0	0	0	0	0	0	0
0	0	0	0	0	0	0	0	1	0
0	0	0	0	0	0	0	0	0	0
55	6	4	6	1	13	0	3	8	1
0	0	0	0	0	0	0	0	0	0
0	0	0	0	0	1	0	0	1	0
0	0	2	1	0	0	0	0	0	0
887	570	937	819	739	973	411	2	1	0
262	828	423	356	218	525	104	1	2	0
329	6	120	144	537	181	1	63	54	17
0	0	0	0	1	0	0	0	0	0
0	0	0	0	44	0	0	0	0	0
0	0	0	0	0	0	0	0	0	0
0	0	0	2	1	3	0	0	0	0
0	0	0	0	0	0	0	0	0	0
0	0	0	1	0	0	0	0	0	0
0	0	0	0	0	0	0	0	0	0
0	0	0	0	0	0	0	0	0	0
0	0	0	0	0	0	0	0	0	0
0	0	1	0	0	0	0	0	0	0
136	89	250	109	32	139	80	1	4	23
0	0	0	0	0	0	0	0	0	0
0	0	0	0	0	0	0	0	0	0
0	0	0	0	0	1	0	0	1	0
0	0	1	0	0	0	0	0	0	0
0	0	0	0	0	0	0	30	36	0
1	0	0	0	1	10	0	1	2	0
9	2	27	18	3	27	0	16	13	6
1	0	0	0	0	0	0	0	0	0
0	0	0	0	0	0	0	0	0	0
0	0	0	0	0	0	0	0	0	0
51	31	141	61	66	90	13	52	71	169
0	0	0	0	0	2	0	1	2	0
0	0	0	0	0	0	0	0	0	0
7	6	0	3	11	4	0	10	12	26
270	244	310	259	156	220	81	128	125	200

289	351	487	323	280	312	107	118	141	294
0	0	0	0	0	0	0	0	0	0
4	1	7	3	0	3	1	1	0	0
0	1	0	0	2	0	0	0	0	0
0	1	0	3	0	3	0	0	3	0
7	0	0	0	0	0	0	0	0	0
0	0	0	0	0	1	0	0	3	0
0	0	0	0	0	0	0	0	0	0
0	0	0	0	0	0	0	0	0	0
0	4	1	42	0	26	0	0	0	0
0	0	0	0	0	0	0	0	0	0
0	0	0	0	0	0	0	0	0	0
0	0	0	0	0	0	0	0	0	0
8	30	19	46	5	16	0	57	24	62
0	1	0	0	0	0	0	0	0	0
0	0	0	0	0	0	0	0	1	0
0	0	0	0	0	0	0	0	0	0
0	0	0	0	0	0	0	0	0	0
0	0	0	0	0	0	0	0	0	0
55	6	4	6	1	13	0	3	8	1
0	0	0	0	0	0	0	0	0	0
0	0	0	0	0	1	0	0	1	0
0	0	2	1	0	0	0	0	0	0
0	1	0	4	4	6	0	2	1	0
887	569	937	815	735	967	411	0	0	0
133	672	396	188	168	458	92	1	1	0
129	156	27	167	49	62	12	0	1	0
0	0	0	1	1	5	0	0	0	0
329	6	120	144	537	181	1	63	54	17
0	0	0	0	1	0	0	0	0	0
0	0	0	0	44	0	0	0	0	0
0	0	0	0	0	0	0	0	0	0
0	0	0	0	0	0	0	0	0	0
0	0	0	2	1	3	0	0	0	0
0	0	0	0	0	0	0	0	0	0
0	0	0	1	0	0	0	0	0	0
0	0	0	0	0	0	0	0	0	0
0	0	0	0	0	0	0	0	0	0

0	0	0	0	0	0	0	0	0	0
0	0	1	0	0	0	0	0	0	0
0	0	0	0	0	0	0	0	0	0
136	89	250	109	32	139	80	1	4	23
0	0	0	0	0	0	0	0	0	0
0	0	0	0	0	0	0	0	0	0
0	0	0	0	0	1	0	0	1	0
0	0	1	0	0	0	0	0	0	0
0	0	0	0	0	0	0	30	36	0
0	0	0	0	0	0	0	0	0	0
0	0	0	0	0	10	0	0	2	0
1	0	0	0	1	0	0	1	0	0
0	0	0	0	0	0	0	0	0	0
9	2	27	18	3	27	0	16	13	6
1	0	0	0	0	0	0	0	0	0
0	0	0	0	0	0	0	0	0	0
0	0	0	0	0	0	0	0	0	0
51	31	141	61	66	90	13	52	71	169
0	0	0	0	0	2	0	1	2	0
0	0	0	0	0	0	0	0	0	0
7	6	0	3	7	3	0	8	12	26
0	0	0	0	4	1	0	2	0	0
1	0	1	0	0	0	0	2	0	1
269	244	309	259	156	220	81	126	125	199
0	0	4	3	0	0	0	0	0	0
0	0	0	0	0	0	0	0	0	0
289	351	482	320	280	311	107	117	137	294
0	0	0	0	0	0	0	0	0	0
0	0	1	0	0	1	0	1	4	0
0	0	0	0	0	0	0	0	0	0
4	1	7	3	0	3	1	1	0	0
0	1	0	0	2	0	0	0	0	0
0	1	0	3	0	3	0	0	3	0
7	0	0	0	0	0	0	0	0	0
0	0	0	0	0	0	0	0	0	0
0	0	0	0	0	1	0	0	3	0
0	0	0	0	0	0	0	0	0	0
0	0	0	0	0	0	0	0	0	0

0	4	1	42	0	26	0	0	0	0
0	0	0	0	0	0	0	0	0	0
0	0	0	0	0	0	0	0	0	0
0	0	0	0	0	0	0	0	0	0
8	29	19	44	5	16	0	56	24	62
0	1	0	2	0	0	0	1	0	0
0	1	0	0	0	0	0	0	0	0
0	0	0	0	0	0	0	0	1	0
0	0	0	0	0	0	0	0	0	0
0	0	0	0	0	0	0	0	0	0
0	0	0	0	0	0	0	0	0	0
0	0	0	0	0	0	0	0	0	0
0	0	0	0	0	0	0	0	0	0
55	6	4	6	1	13	0	3	8	1
0	0	0	0	0	0	0	0	0	0
0	0	0	0	0	0	0	0	0	0
0	0	0	0	0	0	0	0	0	0
0	0	0	0	0	1	0	0	1	0
0	0	2	1	0	0	0	0	0	0
0	0	0	0	1	0	0	0	0	0
0	1	0	4	3	6	0	2	1	0
887	569	937	815	735	967	411	0	0	0
133	672	396	188	168	458	92	1	1	0
129	156	27	167	49	62	12	0	1	0
0	0	0	1	1	5	0	0	0	0
0	0	0	0	0	0	0	0	0	0
28	6	0	13	228	4	0	0	0	0
0	0	0	0	0	4	0	0	1	0
38	0	86	1	0	0	0	0	0	0
0	0	0	2	1	3	0	3	2	0
1	0	0	0	0	1	0	0	0	0
180	0	0	69	38	154	1	13	12	5
0	0	0	0	0	0	0	0	0	0
2	0	0	9	5	5	0	11	8	4
68	0	33	43	191	1	0	10	9	0
2	0	0	0	0	1	0	1	1	0
0	0	1	0	0	0	0	0	0	0
0	0	0	2	5	0	0	0	3	8

1	0	0	0	1	0	0	0	0	0
0	0	0	0	0	0	0	0	0	0
0	0	0	0	0	0	0	0	1	0
0	0	0	0	18	0	0	0	0	0
0	0	0	0	0	0	0	0	0	0
1	0	0	1	0	2	0	0	1	0
2	0	0	3	41	3	0	3	2	0
1	0	0	0	0	0	0	0	0	0
5	0	0	1	9	3	0	0	0	0
0	0	0	0	0	0	0	22	14	0
0	0	0	0	1	0	0	0	0	0
0	0	0	0	44	0	0	0	0	0
0	0	0	0	0	0	0	0	0	0
0	0	0	0	0	0	0	0	0	0
0	0	0	0	0	0	0	0	0	0
0	0	0	0	0	0	0	0	0	0
0	0	0	2	1	3	0	0	0	0
0	0	0	0	0	0	0	0	0	0

bbsdi7	wbmdi9	wbmdi4	bbmdi6	bbsdi8	wbmdi2	wbsdi5	bbsdi1	bbmdi5	bbmdi11
0	0	0	0	0	0	0	0	0	0
729	1299	159	980	315	119	353	745	493	375
0	0	0	0	0	0	0	0	0	0
7	37	0	4	1	1	1	12	4	1
0	0	0	0	0	0	1	0	0	0
0	6	0	0	0	0	0	0	0	0
82	6	3	72	26	5	23	38	43	40
466	1153	139	645	211	108	218	582	337	236
173	97	17	258	77	5	110	113	109	98
1	0	0	1	0	0	0	0	0	0
0	0	0	0	0	0	0	0	0	0
7	37	0	4	1	1	1	12	4	1
0	0	0	0	0	0	1	0	0	0
0	0	0	0	0	0	0	0	0	0
0	6	0	0	0	0	0	0	0	0
0	0	0	0	0	0	0	0	0	0
82	6	3	72	26	5	23	38	43	40
466	1153	139	645	211	108	218	582	337	236
0	0	0	0	0	0	0	0	0	0
85	39	1	62	33	0	59	48	58	54
8	0	0	7	0	0	9	5	2	2
0	0	0	0	0	0	0	0	0	0
80	58	16	189	44	5	42	60	49	42
1	0	0	1	0	0	0	0	0	0
0	0	0	0	0	0	0	0	0	0
1	0	0	1	0	0	1	1	1	0
6	37	0	3	1	1	0	11	3	1
0	0	0	0	0	0	1	0	0	0
0	0	0	0	0	0	0	0	0	0
0	6	0	0	0	0	0	0	0	0
0	0	0	0	0	0	0	0	0	0
82	6	3	72	26	5	23	38	43	40
83	238	39	149	35	14	50	124	61	42
382	915	100	494	176	94	168	456	275	194
1	0	0	2	0	0	0	2	1	0
0	0	0	0	0	0	0	0	0	0
0	0	0	0	0	0	0	0	0	0

0	1	0	0	0	0	0	0	1	0
85	38	1	62	33	0	59	48	57	54
0	0	0	0	0	0	0	0	0	0
0	0	0	1	0	0	0	0	0	0
0	0	0	0	0	0	0	0	0	0
8	0	0	6	0	0	9	4	2	2
0	0	0	0	0	0	0	1	0	0
0	0	0	0	0	0	0	0	0	0
0	0	0	0	0	0	0	0	0	0
2	0	0	3	3	0	1	1	2	1
0	0	0	1	2	0	0	0	0	1
73	58	15	183	39	5	41	59	47	39
0	0	0	0	0	0	0	0	0	0
5	0	0	2	0	0	0	0	0	1
0	0	1	0	0	0	0	0	0	0
0	0	0	0	0	0	0	0	0	0
1	0	0	1	0	0	0	0	0	0
0	0	0	0	0	0	0	0	0	0
0	0	0	1	0	0	0	0	0	0
0	0	0	0	0	0	0	0	0	0
0	0	0	0	0	0	0	0	0	0
1	0	0	0	0	0	1	1	1	0
6	37	0	3	1	1	0	11	3	1
0	0	0	0	0	0	1	0	0	0
0	0	0	0	0	0	0	0	0	0
0	6	0	0	0	0	0	0	0	0
0	0	0	0	0	0	0	0	0	0
82	6	3	72	26	5	23	38	43	40
1	0	0	3	0	0	0	1	1	0
16	23	14	18	2	3	10	21	11	9
0	0	0	0	0	0	0	1	0	0
0	0	0	0	0	0	0	0	0	0
0	0	0	1	0	0	0	0	0	0
65	215	25	126	33	11	40	101	49	33
1	0	0	1	0	0	0	0	0	0
0	0	0	0	0	0	1	0	0	0
12	16	2	20	5	31	2	13	6	4
169	481	59	230	74	21	72	194	143	68

197	416	39	244	92	42	92	249	123	121
0	0	0	0	2	0	0	0	2	0
3	1	0	0	2	0	1	0	1	1
1	1	0	0	1	0	0	0	0	0
1	0	0	2	0	0	0	2	1	0
0	0	0	0	0	0	0	0	0	0
0	0	0	0	0	0	0	0	0	0
0	0	0	0	0	0	0	0	0	0
0	0	0	0	0	0	0	0	0	0
0	1	0	0	0	0	0	0	0	0
0	0	0	0	0	0	0	0	1	0
0	0	0	0	0	0	0	0	0	0
0	0	0	0	0	0	0	0	0	0
85	38	1	62	33	0	59	48	57	54
0	0	0	0	0	0	0	0	0	0
0	0	0	0	0	0	0	0	0	0
0	0	0	1	0	0	0	0	0	0
0	0	0	0	0	0	0	0	0	0
0	0	0	0	0	0	0	0	0	0
8	0	0	6	0	0	9	4	2	2
0	0	0	0	0	0	0	1	0	0
0	0	0	0	0	0	0	0	0	0
0	0	0	0	0	0	0	0	0	0
0	0	0	1	1	0	0	0	0	0
2	0	0	2	2	0	1	1	2	1
0	0	0	1	1	0	0	0	0	1
0	0	0	0	1	0	0	0	0	0
0	0	0	0	0	0	0	0	0	0
73	58	15	183	39	5	41	59	47	39
0	0	0	0	0	0	0	0	0	0
0	0	0	0	0	0	0	0	0	1
5	0	0	2	0	0	0	0	0	0
0	0	1	0	0	0	0	0	0	0
0	0	0	0	0	0	0	0	0	0
1	0	0	1	0	0	0	0	0	0
0	0	0	0	0	0	0	0	0	0
0	0	0	1	0	0	0	0	0	0
0	0	0	0	0	0	0	0	0	0

0	0	0	0	0	0	0	0	0	0
0	0	0	0	0	0	0	0	0	0
1	0	0	0	0	0	1	1	1	0
6	37	0	3	1	1	0	11	3	1
0	0	0	0	0	0	1	0	0	0
0	0	0	0	0	0	0	0	0	0
0	6	0	0	0	0	0	0	0	0
0	0	0	0	0	0	0	0	0	0
82	6	3	72	26	5	23	38	43	40
1	0	0	0	0	0	0	0	0	0
0	0	0	3	0	0	0	1	0	0
0	0	0	0	0	0	0	0	1	0
0	0	0	0	0	0	0	0	0	0
16	23	14	18	2	3	10	21	11	9
0	0	0	0	0	0	0	1	0	0
0	0	0	0	0	0	0	0	0	0
0	0	0	1	0	0	0	0	0	0
65	215	25	126	33	11	40	101	49	33
1	0	0	1	0	0	0	0	0	0
0	0	0	0	0	0	1	0	0	0
11	16	1	20	5	31	2	13	6	4
1	0	1	0	0	0	0	0	0	0
1	1	0	3	3	0	0	5	2	2
168	480	59	227	71	21	72	189	141	66
1	0	0	0	0	0	0	0	0	0
0	0	0	0	0	0	0	0	0	0
195	416	39	242	91	42	92	245	121	120
0	0	0	1	0	0	0	0	0	0
1	0	0	1	1	0	0	4	2	1
0	0	0	0	2	0	0	0	2	0
3	1	0	0	2	0	1	0	1	1
1	1	0	0	1	0	0	0	0	0
1	0	0	2	0	0	0	2	1	0
0	0	0	0	0	0	0	0	0	0
0	0	0	0	0	0	0	0	0	0
0	0	0	0	0	0	0	0	0	0
0	0	0	0	0	0	0	0	0	0
0	0	0	0	0	0	0	0	0	0

0	1	0	0	0	0	0	0	0	0
0	0	0	0	0	0	0	0	1	0
0	0	0	0	0	0	0	0	0	0
0	0	0	0	0	0	0	0	0	0
85	38	1	59	31	0	59	48	54	54
0	0	0	3	2	0	0	0	3	0
0	0	0	0	0	0	0	0	0	0
0	0	0	0	0	0	0	0	0	0
0	0	0	0	0	0	0	0	0	0
0	0	0	0	0	0	0	0	0	0
0	0	0	1	0	0	0	0	0	0
0	0	0	0	0	0	0	0	0	0
0	0	0	0	0	0	0	0	0	0
8	0	0	6	0	0	7	4	2	2
0	0	0	0	0	0	1	0	0	0
0	0	0	0	0	0	1	0	0	0
0	0	0	0	0	0	0	1	0	0
0	0	0	0	0	0	0	0	0	0
0	0	0	0	0	0	0	0	0	0
0	0	0	0	0	0	0	0	0	0
0	0	0	1	1	0	0	0	0	0
2	0	0	2	2	0	1	1	2	1
0	0	0	1	1	0	0	0	0	1
0	0	0	0	1	0	0	0	0	0
0	0	0	0	0	0	0	0	0	0
0	0	0	0	1	0	0	1	1	0
0	1	0	0	0	0	0	0	0	0
0	1	0	0	0	0	0	0	0	0
1	0	0	1	0	3	0	0	0	2
0	0	0	1	1	0	0	0	0	0
0	0	0	0	0	0	0	0	0	0
22	49	12	21	8	0	6	17	15	9
0	0	0	0	0	0	0	0	0	0
8	0	0	12	5	1	10	5	9	5
12	3	2	12	8	1	3	8	2	4
2	0	0	5	0	0	0	1	0	2
0	0	0	0	0	0	0	0	0	0
3	0	0	5	1	0	1	7	3	5

0	0	0	2	1	0	0	0	0	0
0	0	0	0	0	0	0	0	0	0
1	0	0	0	0	0	0	0	0	0
0	0	0	0	0	0	0	1	0	0
0	0	0	0	0	0	0	0	0	0
0	0	0	0	0	0	0	1	0	0
3	2	1	2	2	0	2	2	1	0
0	1	0	0	0	0	0	0	0	0
0	1	0	1	0	0	0	0	1	0
21	0	0	121	12	0	19	16	15	12
0	0	0	0	0	0	0	0	0	0
0	0	0	0	0	0	0	0	0	1
5	0	0	2	0	0	0	0	0	0
0	0	0	0	0	0	0	0	0	0
0	0	1	0	0	0	0	0	0	0
0	0	0	0	0	0	0	0	0	0
0	0	0	0	0	0	0	0	0	0
1	0	0	1	0	0	0	0	0	0

wbmdi1	wbsdi4	bbsdi9	wbsdi10	bbmdi9	bbmdi2	bbsdi6	bbmdi10	wbsdi7	bbmdi8	
0	0	0	0	0	0	0	0	0	0	0
433	116	299	33	1124	469	476	486	58	665	
0	0	0	0	0	0	0	0	0	0	
14	3	1	1	19	0	0	1	0	5	
0	0	0	0	0	0	0	0	0	0	
0	0	0	0	0	0	0	0	0	0	
8	2	22	0	80	24	31	55	0	54	
383	99	210	23	842	329	335	289	49	472	
28	12	66	9	182	116	110	141	9	134	
0	0	0	0	1	0	0	0	0	0	
0	0	0	0	0	0	0	0	0	0	
14	3	1	1	19	0	0	1	0	5	
0	0	0	0	0	0	0	0	0	0	
0	0	0	0	0	0	0	0	0	0	
0	0	0	0	0	0	0	0	0	0	
0	0	0	0	0	0	0	0	0	0	
8	2	22	0	80	24	31	55	0	54	
383	99	210	23	842	329	333	289	49	472	
0	0	0	0	0	0	2	0	0	0	
20	1	18	4	115	49	49	68	9	52	
0	8	3	1	9	6	4	15	0	4	
0	0	0	0	0	0	0	0	0	0	
8	3	45	4	58	61	57	58	0	78	
0	0	0	0	1	0	0	0	0	0	
0	0	0	0	0	0	0	0	0	0	
0	0	0	0	1	0	0	0	0	0	
14	3	1	1	18	0	0	1	0	5	
0	0	0	0	0	0	0	0	0	0	
0	0	0	0	0	0	0	0	0	0	
0	0	0	0	0	0	0	0	0	0	
0	0	0	0	0	0	0	0	0	0	
8	2	22	0	80	24	31	55	0	54	
52	16	32	4	159	77	53	63	11	80	
331	83	178	19	683	252	280	226	38	391	
0	0	0	0	0	0	0	0	0	1	
0	0	0	0	0	0	2	0	0	0	
0	0	0	0	0	1	0	0	0	0	

0	0	0	2	0	0	0	2	0	0
20	1	18	2	115	48	49	66	9	52
0	0	0	0	0	0	0	0	0	0
0	0	0	0	0	1	1	2	0	0
0	0	0	0	0	0	1	1	0	0
0	8	3	1	9	5	2	12	0	4
0	0	0	0	0	0	0	0	0	0
0	0	0	0	0	0	0	0	0	0
0	0	0	0	0	0	0	0	0	0
1	0	1	0	1	1	0	1	0	0
0	0	0	0	0	0	0	0	0	1
7	1	43	4	57	59	54	56	0	76
0	0	0	0	0	0	0	0	0	0
0	2	1	0	0	0	2	1	0	0
0	0	0	0	0	0	0	0	0	1
0	0	0	0	0	1	1	0	0	0
0	0	0	0	1	0	0	0	0	0
0	0	0	0	0	0	0	0	0	0
0	0	0	0	0	0	0	0	0	0
0	0	0	0	0	0	0	0	0	0
0	0	0	0	0	0	0	0	0	0
0	0	0	0	1	0	0	0	0	0
14	3	1	1	18	0	0	1	0	5
0	0	0	0	0	0	0	0	0	0
0	0	0	0	0	0	0	0	0	0
0	0	0	0	0	0	0	0	0	0
0	0	0	0	0	0	0	0	0	0
8	2	22	0	80	24	31	55	0	54
0	0	0	0	0	1	0	1	0	2
7	6	5	2	21	10	6	8	2	8
0	0	0	0	0	0	0	0	0	0
0	0	0	0	0	0	0	0	0	0
0	0	0	0	0	0	1	0	0	0
45	10	27	2	137	65	46	54	9	70
0	0	0	0	1	1	0	0	0	0
0	0	0	0	0	0	0	0	0	0
7	7	9	0	21	13	6	6	3	9
151	40	72	6	336	106	124	105	6	187

173	36	94	13	323	131	150	115	29	193
0	0	1	0	2	0	0	0	0	0
0	0	2	0	1	2	0	0	0	2
0	0	0	0	0	0	0	0	0	0
0	0	0	0	0	0	0	0	0	1
0	0	0	0	0	0	0	0	0	0
0	0	0	0	0	0	2	0	0	0
0	0	0	0	0	0	0	0	0	0
0	0	0	0	0	1	0	0	0	0
0	0	0	2	0	0	0	1	0	0
0	0	0	0	0	0	0	0	0	0
0	0	0	0	0	0	0	1	0	0
0	0	0	0	0	0	0	0	0	0
20	1	18	2	115	48	49	66	9	52
0	0	0	0	0	0	0	0	0	0
0	0	0	0	0	0	0	0	0	0
0	0	0	0	0	0	1	2	0	0
0	0	0	0	0	1	0	0	0	0
0	0	0	0	0	0	1	1	0	0
0	8	3	1	9	5	2	12	0	4
0	0	0	0	0	0	0	0	0	0
0	0	0	0	0	0	0	0	0	0
0	0	0	0	0	0	0	0	0	0
0	0	0	0	0	0	0	0	0	0
1	0	1	0	1	1	0	1	0	0
0	0	0	0	0	0	0	0	0	1
0	0	0	0	0	0	0	0	0	0
0	0	0	0	0	0	0	0	0	0
7	1	43	4	57	59	54	56	0	76
0	0	0	0	0	0	0	0	0	0
0	0	0	0	0	0	0	0	0	0
0	2	1	0	0	0	2	1	0	0
0	0	0	0	0	0	0	0	0	1
0	0	0	0	0	1	1	0	0	0
0	0	0	0	1	0	0	0	0	0
0	0	0	0	0	0	0	0	0	0
0	0	0	0	0	0	0	0	0	0
0	0	0	0	0	0	0	0	0	0

0	0	0	0	0	0	0	0	0	0
0	0	0	0	0	0	0	0	0	0
0	0	0	0	1	0	0	0	0	0
14	3	1	1	18	0	0	1	0	5
0	0	0	0	0	0	0	0	0	0
0	0	0	0	0	0	0	0	0	0
0	0	0	0	0	0	0	0	0	0
0	0	0	0	0	0	0	0	0	0
8	2	22	0	80	24	31	55	0	54
0	0	0	0	0	0	0	0	0	0
0	0	0	0	0	0	0	1	0	2
0	0	0	0	0	1	0	0	0	0
0	0	0	0	0	0	0	0	0	0
7	6	5	2	21	10	6	8	2	8
0	0	0	0	0	0	0	0	0	0
0	0	0	0	0	0	0	0	0	0
0	0	0	0	0	0	1	0	0	0
45	10	27	2	137	65	46	54	9	70
0	0	0	0	1	1	0	0	0	0
0	0	0	0	0	0	0	0	0	0
7	7	9	0	21	13	6	6	3	9
0	0	0	0	0	0	0	0	0	0
0	0	1	0	4	4	1	2	0	0
151	40	71	6	332	102	123	103	6	187
0	0	0	0	2	0	0	0	0	1
0	0	0	0	0	0	0	0	0	0
172	36	91	13	320	130	150	115	29	191
0	0	0	0	0	0	0	0	0	0
1	0	3	0	1	1	0	0	0	1
0	0	1	0	2	0	0	0	0	0
0	0	2	0	1	2	0	0	0	2
0	0	0	0	0	0	0	0	0	0
0	0	0	0	0	0	0	0	0	1
0	0	0	0	0	0	0	0	0	0
0	0	0	0	0	0	0	0	0	0
0	0	0	0	0	0	2	0	0	0
0	0	0	0	0	0	0	0	0	0
0	0	0	0	0	1	0	0	0	0

0	0	0	2	0	0	0	1	0	0
0	0	0	0	0	0	0	0	0	0
0	0	0	0	0	0	0	1	0	0
0	0	0	0	0	0	0	0	0	0
20	1	18	2	114	45	49	65	9	51
0	0	0	0	1	3	0	1	0	1
0	0	0	0	0	0	0	0	0	0
0	0	0	0	0	0	0	0	0	0
0	0	0	0	0	0	0	1	0	0
0	0	0	0	0	0	0	1	0	0
0	0	0	0	0	0	1	0	0	0
0	0	0	0	0	1	0	0	0	0
0	0	0	0	0	0	1	1	0	0
0	8	3	1	9	5	2	12	0	4
0	0	0	0	0	0	0	0	0	0
0	0	0	0	0	0	0	0	0	0
0	0	0	0	0	0	0	0	0	0
0	0	0	0	0	0	0	0	0	0
0	0	0	0	0	0	0	0	0	0
0	0	0	0	0	0	0	0	0	0
0	0	0	0	0	0	0	0	0	0
1	0	1	0	1	1	0	1	0	0
0	0	0	0	0	0	0	0	0	1
0	0	0	0	0	0	0	0	0	0
0	0	0	0	0	0	0	0	0	0
0	1	0	0	0	0	0	0	0	0
0	0	0	0	0	0	0	0	0	1
0	0	0	0	0	0	0	0	0	0
1	0	0	0	0	1	3	1	0	2
0	0	0	0	2	1	0	1	0	0
0	0	0	0	0	0	0	0	0	0
5	0	16	0	33	14	13	10	0	17
0	0	0	0	0	0	0	0	0	0
0	0	4	0	5	8	8	18	0	15
0	0	4	4	7	7	7	3	0	9
0	0	0	0	0	0	1	0	0	1
0	0	0	0	0	0	0	0	0	0
0	0	4	0	2	2	4	1	0	1

0	0	0	0	0	0	0	1	0	0
0	0	0	0	0	0	0	0	0	0
0	0	0	0	0	0	0	0	0	0
0	0	1	0	0	0	0	0	0	0
0	0	1	0	0	0	0	0	0	0
0	0	0	0	0	0	0	0	0	0
1	0	1	0	0	0	1	3	0	4
0	0	0	0	0	0	0	0	0	0
0	0	0	0	0	0	0	0	0	0
0	0	12	0	8	26	17	18	0	26
0	0	0	0	0	0	0	0	0	0
0	0	0	0	0	0	0	0	0	0
0	2	1	0	0	0	2	1	0	0
0	0	0	0	0	0	0	0	0	1
0	0	0	0	0	0	0	0	0	0
0	0	0	0	0	0	0	0	0	0
0	0	0	0	0	1	1	0	0	0
0	0	0	0	1	0	0	0	0	0

wbsdi2	wbsdi8	wbmdi10	bbmdi4	wbmdi6	wbmdi3	wbsdi9	bbmdi7	bbmdi1	wbmdi5
0	0	0	0	0	0	0	0	0	0
270	371	31	482	190	20	55	714	106	80
0	0	0	0	0	0	0	0	0	0
0	5	1	9	1	1	2	4	3	0
0	0	0	0	0	0	0	0	0	0
0	3	0	0	1	0	1	0	0	0
20	0	0	34	8	0	1	138	3	1
161	187	28	324	142	18	46	457	91	53
89	176	2	115	38	1	5	115	9	26
0	0	0	0	0	0	0	0	0	0
0	0	0	0	0	0	0	0	0	0
0	5	1	9	1	1	2	4	3	0
0	0	0	0	0	0	0	0	0	0
0	0	0	0	0	0	0	0	0	0
0	3	0	0	1	0	1	0	0	0
0	0	0	0	0	0	0	0	0	0
20	0	0	34	8	0	1	138	3	1
161	187	28	324	142	18	46	457	91	53
0	0	0	0	0	0	0	0	0	0
27	72	1	64	7	1	1	45	6	1
10	2	1	4	0	0	1	8	0	0
0	0	0	0	0	0	0	0	0	0
52	102	0	47	31	0	3	62	3	25
0	0	0	0	0	0	0	0	0	0
0	0	0	0	0	0	0	0	0	0
0	0	1	1	0	0	0	1	0	0
0	5	0	8	1	1	2	3	3	0
0	0	0	0	0	0	0	0	0	0
0	0	0	0	0	0	0	0	0	0
0	3	0	0	1	0	1	0	0	0
0	0	0	0	0	0	0	0	0	0
20	0	0	34	8	0	1	138	3	1
29	21	8	71	34	5	5	102	11	6
132	166	20	252	108	13	41	353	80	47
0	0	0	1	0	0	0	2	0	0
0	0	0	0	0	0	0	0	0	0
0	0	0	0	0	0	0	0	0	0

0	0	0	0	0	0	0	0	0	0
27	72	1	64	7	1	1	45	6	1
0	0	0	0	0	0	0	0	0	0
0	0	0	0	0	0	1	0	0	0
0	0	0	0	0	0	0	0	0	0
10	2	1	4	0	0	0	8	0	0
0	0	0	0	0	0	0	0	0	0
0	0	0	0	0	0	0	0	0	0
0	0	0	0	0	0	0	0	0	0
0	0	0	2	0	0	0	0	1	0
0	1	0	0	0	0	0	0	0	0
52	101	0	45	31	0	3	62	2	25
0	0	0	0	0	0	0	0	0	0
0	0	0	0	0	0	0	0	0	0
0	0	0	0	0	0	0	0	0	0
0	0	0	0	0	0	0	0	0	0
0	0	0	0	0	0	0	0	0	0
0	0	0	0	0	0	0	0	0	0
0	0	0	0	0	0	0	0	0	0
0	0	1	0	0	0	0	0	0	0
0	0	0	0	0	0	0	1	0	0
0	0	0	1	0	0	0	0	0	0
0	5	0	8	1	1	2	3	3	0
0	0	0	0	0	0	0	0	0	0
0	0	0	0	0	0	0	0	0	0
0	3	0	0	1	0	1	0	0	0
0	0	0	0	0	0	0	0	0	0
20	0	0	34	8	0	1	138	3	1
0	0	0	0	0	0	0	1	0	0
3	4	1	20	3	1	0	15	2	3
0	0	0	0	0	0	0	0	0	0
0	2	0	0	0	0	0	2	0	0
0	0	0	0	0	0	0	0	0	0
26	15	7	51	31	4	5	84	9	3
0	0	0	0	0	0	0	0	0	0
0	0	0	0	0	0	0	0	0	0
4	3	1	9	0	0	1	14	2	5
51	70	10	137	50	5	11	162	24	17

77	93	9	105	57	7	29	177	54	25
0	0	0	0	0	0	0	0	0	0
0	0	0	1	1	1	0	0	0	0
0	0	0	0	0	0	0	0	0	0
0	0	0	1	0	0	0	2	0	0
0	0	0	0	0	0	0	0	0	0
0	0	0	0	0	0	0	0	0	0
0	0	0	0	0	0	0	0	0	0
0	0	0	0	0	0	0	0	0	0
0	0	0	0	0	0	0	0	0	0
0	0	0	0	0	0	0	0	0	0
0	0	0	0	0	0	0	0	0	0
0	0	0	0	0	0	0	0	0	0
27	72	1	64	7	1	1	45	6	1
0	0	0	0	0	0	0	0	0	0
0	0	0	0	0	0	0	0	0	0
0	0	0	0	0	0	0	0	0	0
0	0	0	0	0	0	1	0	0	0
0	0	0	0	0	0	0	0	0	0
10	2	1	4	0	0	0	8	0	0
0	0	0	0	0	0	0	0	0	0
0	0	0	0	0	0	0	0	0	0
0	0	0	0	0	0	0	0	0	0
0	0	0	2	0	0	0	0	0	0
0	0	0	0	0	0	0	0	1	0
0	1	0	0	0	0	0	0	0	0
0	0	0	0	0	0	0	0	0	0
0	0	0	0	0	0	0	0	0	0
52	101	0	45	31	0	3	62	2	25
0	0	0	0	0	0	0	0	0	0
0	0	0	0	0	0	0	0	0	0
0	0	0	0	0	0	0	0	0	0
0	0	0	0	0	0	0	0	0	0
0	0	0	0	0	0	0	0	0	0
0	0	0	0	0	0	0	0	0	0
0	0	0	0	0	0	0	0	0	0
0	0	0	0	0	0	0	0	0	0
0	0	1	0	0	0	0	0	0	0

0	0	0	0	0	0	0	1	0	0
0	0	0	0	0	0	0	0	0	0
0	0	0	1	0	0	0	0	0	0
0	5	0	8	1	1	2	3	3	0
0	0	0	0	0	0	0	0	0	0
0	0	0	0	0	0	0	0	0	0
0	3	0	0	1	0	1	0	0	0
0	0	0	0	0	0	0	0	0	0
20	0	0	34	8	0	1	138	3	1
0	0	0	0	0	0	0	0	0	0
0	0	0	0	0	0	0	1	0	0
0	0	0	0	0	0	0	0	0	0
0	0	0	0	0	0	0	0	0	0
3	4	1	20	3	1	0	15	2	3
0	0	0	0	0	0	0	0	0	0
0	2	0	0	0	0	0	2	0	0
0	0	0	0	0	0	0	0	0	0
26	15	7	51	31	4	5	84	9	3
0	0	0	0	0	0	0	0	0	0
0	0	0	0	0	0	0	0	0	0
4	3	1	9	0	0	1	14	2	5
0	0	0	0	0	0	0	0	0	0
4	4	1	1	0	0	0	2	0	0
47	66	9	136	50	5	11	160	24	17
1	0	1	0	0	0	0	1	0	0
0	0	0	0	0	0	0	0	0	0
76	93	8	105	57	7	29	175	54	25
0	0	0	0	0	0	0	0	0	0
0	0	0	0	0	0	0	1	0	0
0	0	0	0	0	0	0	0	0	0
0	0	0	1	1	1	0	0	0	0
0	0	0	0	0	0	0	0	0	0
0	0	0	1	0	0	0	2	0	0
0	0	0	0	0	0	0	0	0	0
0	0	0	0	0	0	0	0	0	0
0	0	0	0	0	0	0	0	0	0
0	0	0	0	0	0	0	0	0	0
0	0	0	0	0	0	0	0	0	0

0	0	0	0	0	0	0	0	0	0
0	0	0	0	0	0	0	0	0	0
0	0	0	0	0	0	0	0	0	0
0	0	0	0	0	0	0	0	0	0
26	72	1	63	7	1	1	42	6	1
1	0	0	1	0	0	0	3	0	0
0	0	0	0	0	0	0	0	0	0
0	0	0	0	0	0	0	0	0	0
0	0	0	0	0	0	0	0	0	0
0	0	0	0	0	0	0	0	0	0
0	0	0	0	0	0	0	0	0	0
0	0	0	0	0	0	1	0	0	0
0	0	0	0	0	0	0	0	0	0
10	2	1	4	0	0	0	8	0	0
0	0	0	0	0	0	0	0	0	0
0	0	0	0	0	0	0	0	0	0
0	0	0	0	0	0	0	0	0	0
0	0	0	0	0	0	0	0	0	0
0	0	0	0	0	0	0	0	0	0
0	0	0	0	0	0	0	0	0	0
0	0	0	2	0	0	0	0	0	0
0	0	0	0	0	0	0	0	1	0
0	1	0	0	0	0	0	0	0	0
0	0	0	0	0	0	0	0	0	0
0	0	0	0	0	0	0	0	0	0
0	0	0	0	0	0	0	0	0	0
0	0	0	0	0	0	0	0	0	0
0	0	0	0	0	0	0	0	0	0
8	0	0	0	0	0	0	1	0	0
1	0	0	0	0	0	0	1	0	0
0	0	0	0	0	0	0	0	0	0
10	33	0	14	8	0	1	21	1	22
0	0	0	0	0	0	0	0	0	0
3	26	0	3	8	0	0	7	0	1
15	36	0	5	0	0	2	4	0	2
0	0	0	1	0	0	0	2	0	0
0	0	0	0	0	0	0	0	0	0
0	3	0	4	0	0	0	4	0	0

0	0	0	0	0	0	0	0	0	0
0	0	0	0	0	0	0	0	0	0
0	0	0	0	0	0	0	0	0	0
1	0	0	0	0	0	0	0	0	0
0	0	0	0	0	0	0	0	0	0
0	0	0	1	0	0	0	0	0	0
1	3	0	0	0	0	0	1	1	0
0	0	0	0	0	0	0	0	0	0
1	0	0	0	0	0	0	1	0	0
12	0	0	17	15	0	0	20	0	0
0	0	0	0	0	0	0	0	0	0
0	0	0	0	0	0	0	0	0	0
0	0	0	0	0	0	0	0	0	0
0	0	0	0	0	0	0	0	0	0
0	0	0	0	0	0	0	0	0	0
0	0	0	0	0	0	0	0	0	0
0	0	0	0	0	0	0	0	0	0
0	0	0	0	0	0	0	0	0	0

bbsdi3	wbmdi7	wbmdi8	bbmdi12	wbsdi6	wbsdi3	wbmdi11	wbmdi12	bbmdi3
0	0	0	0	0	0	0	0	0
68	6	20	16	18	32	8	2	5
0	0	0	0	0	0	0	0	0
0	0	0	0	0	1	0	0	0
0	0	0	0	0	0	0	0	0
0	0	0	1	0	0	0	0	0
7	0	1	0	2	0	0	0	1
48	6	14	13	14	23	7	2	4
13	0	5	2	2	8	1	0	0
0	0	0	0	0	0	0	0	0
0	0	0	0	0	0	0	0	0
0	0	0	0	0	1	0	0	0
0	0	0	0	0	0	0	0	0
0	0	0	0	0	0	0	0	0
0	0	0	1	0	0	0	0	0
0	0	0	0	0	0	0	0	0
7	0	1	0	2	0	0	0	1
48	6	14	13	14	23	7	2	4
0	0	0	0	0	0	0	0	0
5	0	0	1	0	1	1	0	0
0	0	0	0	0	0	0	0	0
0	0	0	0	0	0	0	0	0
8	0	5	1	2	7	0	0	0
0	0	0	0	0	0	0	0	0
0	0	0	0	0	0	0	0	0
0	0	0	0	0	0	0	0	0
0	0	0	0	0	1	0	0	0
0	0	0	0	0	0	0	0	0
0	0	0	0	0	0	0	0	0
0	0	0	1	0	0	0	0	0
0	0	0	0	0	0	0	0	0
7	0	1	0	2	0	0	0	1
13	2	2	2	5	5	2	0	1
35	4	12	11	9	18	5	2	3
0	0	0	0	0	0	0	0	0
0	0	0	0	0	0	0	0	0
0	0	0	0	0	0	0	0	0

0	0	0	0	0	0	0	0	0
5	0	0	1	0	1	1	0	0
0	0	0	0	0	0	0	0	0
0	0	0	0	0	0	0	0	0
0	0	0	0	0	0	0	0	0
0	0	0	0	0	0	0	0	0
0	0	0	0	0	0	0	0	0
0	0	0	0	0	0	0	0	0
0	0	0	0	0	0	0	0	0
0	0	0	0	0	0	0	0	0
0	0	0	0	0	0	0	0	0
8	0	5	1	2	7	0	0	0
0	0	0	0	0	0	0	0	0
0	0	0	0	0	0	0	0	0
0	0	0	0	0	0	0	0	0
0	0	0	0	0	0	0	0	0
0	0	0	0	0	0	0	0	0
0	0	0	0	0	0	0	0	0
0	0	0	0	0	0	0	0	0
0	0	0	0	0	0	0	0	0
0	0	0	0	0	0	0	0	0
0	0	0	0	0	0	0	0	0
0	0	0	0	0	1	0	0	0
0	0	0	0	0	0	0	0	0
0	0	0	0	0	0	0	0	0
0	0	0	1	0	0	0	0	0
0	0	0	0	0	0	0	0	0
7	0	1	0	2	0	0	0	1
0	0	0	0	0	0	0	0	0
1	0	0	1	3	0	0	0	0
0	0	0	0	0	0	0	0	0
0	0	0	0	0	0	0	0	0
0	0	0	0	0	0	0	0	0
12	2	2	1	2	5	2	0	1
0	0	0	0	0	0	0	0	0
0	0	0	0	0	0	0	0	0
0	1	0	0	0	0	0	0	0
18	0	3	6	7	6	0	0	3

17	3	8	5	2	12	5	2	0
0	0	1	0	0	0	0	0	0
0	0	0	0	0	0	0	0	0
0	0	0	0	0	0	0	0	0
0	0	0	0	0	0	0	0	0
0	0	0	0	0	0	0	0	0
0	0	0	0	0	0	0	0	0
0	0	0	0	0	0	0	0	0
0	0	0	0	0	0	0	0	0
0	0	0	0	0	0	0	0	0
0	0	0	0	0	0	0	0	0
0	0	0	0	0	0	0	0	0
0	0	0	0	0	0	0	0	0
5	0	0	1	0	1	1	0	0
0	0	0	0	0	0	0	0	0
0	0	0	0	0	0	0	0	0
0	0	0	0	0	0	0	0	0
0	0	0	0	0	0	0	0	0
0	0	0	0	0	0	0	0	0
0	0	0	0	0	0	0	0	0
0	0	0	0	0	0	0	0	0
0	0	0	0	0	0	0	0	0
0	0	0	0	0	0	0	0	0
0	0	0	0	0	0	0	0	0
0	0	0	0	0	0	0	0	0
0	0	0	0	0	0	0	0	0
0	0	0	0	0	0	0	0	0
0	0	0	0	0	0	0	0	0
8	0	5	1	2	7	0	0	0
0	0	0	0	0	0	0	0	0
0	0	0	0	0	0	0	0	0
0	0	0	0	0	0	0	0	0
0	0	0	0	0	0	0	0	0
0	0	0	0	0	0	0	0	0
0	0	0	0	0	0	0	0	0
0	0	0	0	0	0	0	0	0
0	0	0	0	0	0	0	0	0
0	0	0	0	0	0	0	0	0

0	0	0	0	0	0	0	0	0
0	0	0	0	0	0	0	0	0
0	0	0	0	0	0	0	0	0
0	0	0	0	0	1	0	0	0
0	0	0	0	0	0	0	0	0
0	0	0	0	0	0	0	0	0
0	0	0	1	0	0	0	0	0
0	0	0	0	0	0	0	0	0
7	0	1	0	2	0	0	0	1
0	0	0	0	0	0	0	0	0
0	0	0	0	0	0	0	0	0
0	0	0	0	0	0	0	0	0
0	0	0	0	0	0	0	0	0
1	0	0	1	3	0	0	0	0
0	0	0	0	0	0	0	0	0
0	0	0	0	0	0	0	0	0
0	0	0	0	0	0	0	0	0
12	2	2	1	2	5	2	0	1
0	0	0	0	0	0	0	0	0
0	0	0	0	0	0	0	0	0
0	1	0	0	0	0	0	0	0
0	0	0	0	0	0	0	0	0
0	0	0	0	1	0	0	0	0
18	0	3	6	6	6	0	0	3
0	0	0	0	0	0	1	0	0
0	0	0	0	0	0	0	0	0
17	3	8	5	2	12	4	2	0
0	0	0	0	0	0	0	0	0
0	0	0	0	0	0	0	0	0
0	0	1	0	0	0	0	0	0
0	0	0	0	0	0	0	0	0
0	0	0	0	0	0	0	0	0
0	0	0	0	0	0	0	0	0
0	0	0	0	0	0	0	0	0
0	0	0	0	0	0	0	0	0
0	0	0	0	0	0	0	0	0
0	0	0	0	0	0	0	0	0
0	0	0	0	0	0	0	0	0

0	0	0	0	0	0	0	0	0
0	0	0	0	0	0	0	0	0
0	0	0	0	0	0	0	0	0
0	0	0	0	0	0	0	0	0
5	0	0	1	0	1	1	0	0
0	0	0	0	0	0	0	0	0
0	0	0	0	0	0	0	0	0
0	0	0	0	0	0	0	0	0
0	0	0	0	0	0	0	0	0
0	0	0	0	0	0	0	0	0
0	0	0	0	0	0	0	0	0
0	0	0	0	0	0	0	0	0
0	0	0	0	0	0	0	0	0
0	0	0	0	0	0	0	0	0
0	0	0	0	0	0	0	0	0
0	0	0	0	0	0	0	0	0
0	0	0	0	0	0	0	0	0
0	0	0	0	0	0	0	0	0
0	0	0	0	0	0	0	0	0
0	0	0	0	0	0	0	0	0
0	0	0	0	0	0	0	0	0
0	0	0	0	0	0	0	0	0
0	0	0	0	0	0	0	0	0
0	0	0	0	0	0	0	0	0
0	0	0	0	0	0	0	0	0
0	0	0	0	0	0	0	0	0
0	0	0	1	0	0	0	0	0
0	0	0	0	0	0	0	0	0
0	0	0	0	0	0	0	0	0
0	0	0	0	0	0	0	0	0
0	0	0	0	0	0	0	0	0
0	0	0	0	1	1	0	0	0
0	0	0	0	0	0	0	0	0
0	0	3	0	0	0	0	0	0
0	0	0	0	0	5	0	0	0
0	0	0	0	0	0	0	0	0
0	0	0	0	0	0	0	0	0
1	0	1	0	0	0	0	0	0

0	0	0	0	0	0	0	0	0
0	0	0	0	0	0	0	0	0
0	0	0	0	0	0	0	0	0
0	0	0	0	0	0	0	0	0
0	0	0	0	0	0	0	0	0
0	0	0	0	0	0	0	0	0
0	0	0	0	1	1	0	0	0
0	0	0	0	0	0	0	0	0
0	0	1	0	0	0	0	0	0
7	0	0	0	0	0	0	0	0
0	0	0	0	0	0	0	0	0
0	0	0	0	0	0	0	0	0
0	0	0	0	0	0	0	0	0
0	0	0	0	0	0	0	0	0
0	0	0	0	0	0	0	0	0
0	0	0	0	0	0	0	0	0
0	0	0	0	0	0	0	0	0
0	0	0	0	0	0	0	0	0
